# The LEDGF/p75 Integrase Binding Domain Interactome Contributes to the Survival, Clonogenicity, and Tumorsphere Formation of Docetaxel-Resistant Prostate Cancer Cells

**DOI:** 10.3390/cells10102723

**Published:** 2021-10-12

**Authors:** Greisha L. Ortiz-Hernandez, Evelyn S. Sanchez-Hernandez, Pedro T. Ochoa, Catherine C. Elix, Hossam R. Alkashgari, James R. W. McMullen, Ubaldo Soto, Shannalee R. Martinez, Carlos J. Diaz Osterman, Michael Mahler, Sourav Roy, Carlos A. Casiano

**Affiliations:** 1 Center for Health Disparities and Molecular Medicine, Department of Basic Sciences, Loma Linda University School of Medicine, Loma Linda, CA 92350, USA; gortizhernandez@students.llu.edu (G.L.O.-H.); esanchezhernandez@students.llu.edu (E.S.S.-H.); PedroOchoa@students.llu.edu (P.T.O.); celix@llu.edu (C.C.E.); halkashgari@students.llu.edu (H.R.A.); jmcmullen@students.llu.edu (J.R.W.M.); usoto@llu.edu (U.S.); shamartinez@psm.edu (S.R.M.); cjdiaz@psm.edu (C.J.D.O.); 2 Department of Physiology, Faculty of Medicine, University of Jeddah, Jeddah 21577, Saudi Arabia; 3 Department of Research, Werfen, San Diego, CA 92131, USA; mmahler@werfen.com; 4 Border Biomedical Research Center, Department of Biological Sciences, College of Science, University of Texas at El Paso, El Paso, TX 79902, USA; sroy1@utep.edu; 5 Department of Medicine, Loma Linda University School of Medicine, Loma Linda, CA 92350, USA

**Keywords:** autoantibodies, cell survival, chemoresistance, docetaxel, LEDGF/p75, integrase binding domain, IBD interactome, prostate cancer

## Abstract

Patients with prostate cancer (PCa) receiving docetaxel chemotherapy invariably develop chemoresistance. The transcription co-activator lens epithelium-derived growth factor p75 (LEDGF/p75), also known as DFS70 and PSIP1, is upregulated in several human cancers, including PCa and promotes resistance to docetaxel and other drugs. The C-terminal region of LEDGF/p75 contains an integrase binding domain (IBD) that tethers nuclear proteins, including the HIV-1 integrase and transcription factors, to active chromatin to promote viral integration and transcription of cellular survival genes. Here, we investigated the contribution of the LEDGF/p75 IBD interactome to PCa chemoresistance. Quantitative immunoblotting revealed that LEDGF/p75 and its IBD-interacting partners are endogenously upregulated in docetaxel-resistant PCa cell lines compared to docetaxel-sensitive parental cells. Using specific human autoantibodies, we co-immunoprecipitated LEDGF/p75 with its endogenous IBD-interacting partners JPO2, menin, MLL, IWS1, ASK1, and PogZ, as well as transcription factors c-MYC and HRP2, in docetaxel-resistant cells, and confirmed their nuclear co-localization by confocal microscopy. Depletion of LEDGF/p75 and selected interacting partners robustly decreased the survival, clonogenicity, and tumorsphere formation capacity of docetaxel-resistant cells. These results implicate the LEDGF/p75 IBD interactome in PCa chemoresistance and could lead to novel therapeutic strategies targeting this protein complex for the treatment of docetaxel-resistant tumors.

## 1. Introduction

Prostate cancer (PCa) is the most frequently diagnosed cancer among American men, with approximately 248,530 new cases and 34,130 deaths estimated in 2021 in the United States [1]. The current standard of care for metastatic castration prostate cancer (mCRPC), the advanced stage of the disease, is anti-androgen therapy using androgen receptor signaling inhibitors (ARSI) in combination with chemotherapy with the taxane drugs docetaxel (DTX) and cabazitaxel (CBZ) [2]. Despite recent advances in the treatment of mCRPC, PCa still remains incurable due to the activation of multiple mechanisms that promote tumor cell resistance to ARSI and taxane chemotherapy [3,4]. A full understanding of these mechanisms is urgently needed to develop novel and more effective treatments for therapy-resistant PCa.

Emerging evidence indicates that tumors exposed to therapeutic drugs for prolonged periods undergo a reprogramming that results in the expansion of cancer stem cell (CSC) populations that upregulate survival pathways and exhibit therapy resistance [5]. Previously, we reported that the transition of chemosensitive mCRPC cells to taxane resistance is characterized by the activation of a transcriptomic program associated with increased epithelial-to-mesenchymal transition (EMT) and stemness, and upregulation of cancer cell survival proteins, such as lens epithelium-derived growth factor of 75 kD (LEDGF/p75) and c-MYC [6]. In addition, our group and others demonstrated that LEDGF/p75, also known as the dense fine speckled autoantigen of 70 kD (DFS70) and PC4 and SFRS1-interacting protein (PSIP1), is upregulated in PCa and other cancers and contributes to tumor aggressive properties, including chemoresistance [7,8,9,10,11,12,13,14,15]. LEDGF/p75 functions as a transcription coactivator within RNA polymerase II (RNAPII) complexes that promotes cellular survival under environmental stressors, including serum starvation, radiation, and cytotoxic drugs [16,17]. Our group reported previously that its ectopic overexpression in PCa cells confers protection to DTX by attenuating drug-induced lysosomal cell death [8]. The LEDGF/p75 pro-survival functions are likely mediated by interactions with transcription factors to upregulate the expression of stress response, antioxidant, and cancer-related genes [11,12,15,16,17,18,19].

LEDGF/p75 is also the target of a predominantly IgG autoantibody response in subsets of patients with PCa, diverse autoimmune and inflammatory conditions, and apparently healthy individuals, including children and young women [16,20]. This cancer-related protein is broadly relevant to human disease given its documented roles in HIV-AIDS, autoimmunity, and eye diseases [16,17,20,21,22].

LEDGF/p75 is a member of the hepatoma-derived growth factor (HDGF) family, which includes HDGF, HRP2 (also known as HDGF2 or HDGFRP2), HRP3, and HDGFL1, and has been implicated in cancer cell proliferation and survival [23,24]. These proteins share homology at their N-terminal region, which contains a methyl-lysine reading PWWP domain that in LEDGF/p75 is critical for recognition of methylated H3K36me2/3 marks in active chromatin; regulation of its transcriptional and pro-survival activity; and interactions with other proteins, including methyl-CpG-binding protein-2 (MeCP2), splicing factors, and DNA repair proteins [25,26,27,28,29,30].

The C-terminus of LEDGF/p75, implicated in its pro-survival function [26,31,32], is mainly comprised of the integrase binding domain (IBD), which serves as the binding site for the HIV-1 integrase (IN) and is essential for LEDGF/p75-mediated tethering of the IN-viral complex to transcriptionally active chromatin sites in order to facilitate viral integration [21,22]. The IBD overlaps almost perfectly (residues 347–429) with the autoepitope recognized by human anti-LEDGF/p75 autoantibodies and serves as a hub for protein–protein interactions that, in concert with the PWWP domain, facilitate the tethering of transcription factors to RNAPII complexes at transcriptionally active sites [16,20,33,34]. Interestingly, HRP2 also has a C-terminal IBD, which enables this protein to maintain residual HIV-1 integration in LEDGF/p75-depleted cells [35,36]. Because of their extensive structural and functional overlap, LEDGF/p75 and HRP2 are considered paralogs that promote leukemic survival and relieve nucleosome-induced barrier to RNAPII transcription in differentiated cells [37,38].

In addition to HIV-IN, other known LEDGF/p75 IBD-interacting partners include the mixed leukemia lineage histone lysine methyltransferase protein MLL and its interacting partner menin, the c-MYC interacting partner and cell division associated protein JPO2 (R1/CDCA7L/RAM2), the RNAPII-associated and RNA-processing regulator IWS1, the DNA replication and apoptosis signaling associated kinase ASK1, the pogo transposable element and chromatin remodeling protein PogZ, and the RNAPII transcription mediator Med-1 [15,19,33,34]. These LEDGF/p75 interacting partners have a disordered IBD-binding motif (IBM) whose phosphorylation regulates their affinity for the IBD [39]. These IBD protein–protein interactions have been carefully characterized using primarily ectopically overexpressed recombinant proteins and in vitro assays [19,27,34,37,39]. However, studies focusing on the endogenous LEDGF/p75 IBD interactome in a specific cancer context, and particularly in chemoresistance, are scarce.

While various members of the LEDGF/p75 IBD interactome, i.e., MLL, menin, and c-MYC, have been implicated in PCa [40,41], the contribution of this interactome to DTX resistance has not been previously investigated. The aim of this study was to evaluate the hypothesis that the LEDGF/p75 IBD interactome is endogenously upregulated in DTX-resistant cells and contributes to chemoresistance, and that targeting this interactome attenuates the survival and aggressive properties of DTX-resistant cells. In this study, we provide evidence for the novel observations that this interactome is endogenously upregulated in DTX-resistant PCa cells; that LEDGF/p75 interacts as part of an endogenous nuclear complex with JPO2, c-MYC, menin, MLL, ASK, PogZ, IWS1, HRP2, and H3K36me2 in DTX-resistant PCa cells; and that depletion of selected members of this interactome decreases the survival, clonogenicity, and tumorsphere formation capacity of DTX-resistant PCa cells. These results identify the LEDGF/p75 IBD interactome as a novel and potentially attractive target for treating DTX-resistant PCa.

## 2. Materials and Methods

### 2.1. Cell Lines

PCa cell lines PC3, DU145, and 22Rv1 were from the American Type Culture Collection (Manassas, VA, USA, Cat# ATCC-CRL-1435, ATCC-HTB-81, and ATCC-CRL-2505, respectively) and cultured in RPMI-1640 medium (Corning, Corning, NY, USA Cat# 10-040-CM), supplemented with 10% (*v*/*v*) fetal bovine serum (FBS, Genesee Scientific, San Diego, CA, USA, Cat# 25-514), penicillin/streptomycin (Corning, Cat# 30-002-CI), and normocin 1G (Invivogen, San Diego, CA, USA, Cat# NC9390718). Cells were grown under 5% CO_2_ at 37 °C. DTX-resistant (DR) PC3 and DU145 cell lines were developed as indicated previously [6,9] and maintained in the presence of 10 nM DTX (LC Laboratories, Wobun, MA, USA, Cat# D-1000). Short tandem repeat (STR) service provided by ATCC (Cat# ATCC-135-XV) was used to authenticate the cell lines. Mycoplasma testing was conducted at least twice a year using the Lonza MycoAlert^TM^ Mycoplasma Detection Kit (Lonza, Basel, Switzerland, Cat# LT07-218).

### 2.2. Antibodies

Rabbit antibodies targeting the following proteins were acquired from Bethyl Laboratories (Montgomery, TX, USA): LEDGF/p75 (Cat# A300-848A), menin (Cat# A300-105A), JPO2 (Cat# A300-846A), and HRP2 (Cat# A304-314A). Rabbit antibodies targeting the following proteins were from Cell Signaling Technology (Danvers, MA, USA): MDR1 (Cat# 13342), IWS1 (Cat# 5681), c-MYC (Cat# 18583), GR (Cat# 12041S), H3K36me2 (Cat# 2091T), and GAPDH (Cat# 5174). Other rabbit antibodies used were against Med-1/TRAPP220 (Abcam, Waltham, MA, USA, Cat# ab243893), PogZ (Aviva Systems Biology, San Diego, CA, USA, Cat# RP39173-P050) and histone H3 (GeneTex, Irvine, CA, USA, Cat# GTX122148). Mouse monoclonal antibodies included ASK1 (Abnova, Walnut, CA, USA Cat# H00010926-M01) JPO2 (Novus Biologicals, Centennial, CO, USA, Cat# NBP2-46198); and horseradish peroxidase (HRP)-conjugated anti-β-actin (Cell Signaling Technologies, Cat# 12620). Human sera containing antinuclear autoantibodies (ANAs) displaying the characteristic monospecific dense fine speckled (DFS) nuclear immunofluorescence pattern that defines immunoreactivity to LEDGF/p75 [16,20,42], or specific to DNA topoisomerase I (TOPO-1/Scl-70), were from the autoimmune serum collections of Werfen (formerly Inova Diagnostics, San Diego, CA, USA) and the Casiano Laboratory.

### 2.3. Immunoblotting

Whole cell lysates were prepared as described previously [6,9], and their protein concentration was determined using the BioRad DC Protein Assay Kit (Cat# 5000112) to ensure equal loading of proteins separated on individual lanes by SDS-PAGE (NuPAGE 4–12%, Thermo Fisher Scientific, Waltham, MA, USA). Electrophoresis was followed by protein transfer to polyvinyl difluoride membranes (MilliporeSigma, Burlington MA, USA, Cat# IPFL00010). Membranes were blocked with 5% dry milk solution prepared in TBS-T buffer (20 mM Tris-HCL, pH 7.6, 140 mM NaCl, and 0.2% Tween 20) and probed with appropriate primary antibodies. After several washes with TBS-T, membranes were incubated with HRP-conjugated secondary anti-rabbit IgG (Cell Signaling Technology, Danvers, MA, USA, Cat# 7074), anti-mouse IgG (Cell Signaling Cat# 7076), or anti-human IgG (Invitrogen, Cat# A18847). Membranes were then washed with TBS-T, and the protein bands were detected by enhanced chemiluminescence (Thermo Fisher Scientific, Waltham, MA, USA, Cat# 34580). Protein bands from at least 3 independent blots were scanned for each protein of interest, quantified using ImageJ software (National Institutes of Health, Bethesda, MD, USA, Fiji Version 1.44a), and normalized to β-actin or glyceraldehyde 3-phosphate dehydrogenase (GAPDH) loading control protein bands to determine fold upregulation.

### 2.4. Quantitative Real-Time PCR

Quantitative real-time PCR (qPCR) was performed as described previously [27]. Briefly, total RNA was extracted from cultured cells using the RNeasy plus mini kit (QIAGEN, Redwood City, CA, USA). The iScript cDNA synthesis kit (Bio-Rad, Hercules, CA, USA, Cat# 1708891) was used to reverse transcribe RNA (0.5 μg) into cDNA. QPCR was performed in the MyiQ real-time PCR detection system using iQ SYBR Green Supermix (Bio-Rad, Hercules, CA, Cat# 1708880), with appropriate primers, following the manufacturer’s recommendations. Primer sequences for LEDGF/p75, menin, and JPO2 were designed using the Primer3 software or obtained from previously published papers. Primers were commercially synthesized by Integrated DNA Technologies (IDT). GAPDH mRNA was used for normalization. Data were normalized to values of corresponding controls.

### 2.5. MTT Viability Assay and Determination of IC50 Values

DTX-resistant cell lines and their parental, drug-sensitive counterparts were seeded in 96-well plates at a density of 5000 cells per well and then treated with DTX (0–10,000 nM) for up to 72 h in at least three independent experiments, each performed with three biological replicates. After treatments, 3-(4,5-dimethylthiazol-2-yl)-2,5-diphenyltetrazolium bromide (MTT) was added to each well (1 mg/mL), and plates were incubated for 2 h in a 5% CO_2_ incubator at 37°C. Plates were then centrifuged at 1500 rpm for 5 min to avoid loss of floating cells caused by DTX-induced mitotic arrest and cell rounding. Supernatants were discarded, and 100 μL of dimethyl sulfoxide (DMSO, Thermo Fisher Scientific, Waltham, MA, USA, Cat# D128-1) was added to each well. Absorbance was measured at 450 nm using a μQuant Microplate Spectrophotometer (BioTek Instruments, Winooski, VT, USA). Values were normalized to the absorbance obtained for untreated, control cells. Standard error of the mean (SEM) was calculated, and IC50 values were extrapolated using Image J.

### 2.6. Ingenuity Pathway Analysis

This analysis was performed using the QIAGEN’s Ingenuity^®^ Pathway Analysis (IPA) software (https://www.qiagenbioinformatics.com/products/ingenuity-pathway-analysis/, accessed on 17 February 2021). This web-based software application facilitates the pathway analysis and interpretation of various datasets (e.g., gene expression, miRNA, and RNAseq) using different biological factors. In this study, the list of differentially expressed genes (DEGs) in PC3-DR and DU145-DR cells with their respective fold change values were used as input for the IPA software. From the list of core analyses, “Expression analysis” based on the “Expr Fold Change” measurement was selected to analyze the direct and indirect relationships of the various DEGs with respect to different diseases and functions, using “Ingenuity Knowledge Base (Genes Only)” as the reference set. IPA is built on comprehensive, manually curated content of the QIAGEN Knowledge Base, which along with powerful algorithms, helps in the identification of most significant pathways and causal relationships associated with experimental data. This is more powerful than gene set enrichment analysis since the knowledge about the direction of effects, rather than mere associations, is utilized. From the several options available in IPA, the “Canonical pathways” option was selected to elaborately visualize all of the activated and inhibited pathways.

### 2.7. Validation of Human Anti-LEDGF/p75 Autoantibodies

Human DFS sera containing autoantibodies to LEDGF/p75 [42] were evaluated using NOVA Lite HEp-2-ANA slides (Werfen, San Diego, CA, USA). To validate the specificity of these autoantibodies, sera were immunoadsorbed with a recombinant polypeptide corresponding to the entire IBD region as described [42]. Sera diluted at 1:80 in PBS with and without the IBD polypeptide were then evaluated for anti-LEDGF/p75 immunoreactivity using HEp-2 ANA slides. FITC-conjugated secondary antibodies were used at 1:100 dilution for detection of anti-LEDGF/p75 autoantibodies. Image acquisition was performed on a Keyence BZ9000 Biorevo fluorescence microscope.

### 2.8. Co-Immunoprecipitation

Co-immunoprecipitation (co-IP) of endogenous proteins was performed from whole cell lysates using an immunoprecipitation kit (Abcam, Cat# ab206996). Briefly, PC3-DR and DU145-DR cells were grown to confluency (80–90%) for 24 h in 100 mm tissue culture-treated dishes (Genesee Scientific, San Diego, CA, USA, Cat# 25-202), and their viability was assessed to ensure minimal spontaneous cell death prior to IP. Cells were washed twice with ice-cold Dulbecco’s PBS (dPBS), scraped in non-denaturing lysis buffer containing the kit’s protease inhibitor cocktail (PIC) on ice, and collected into pre-chilled 1.5 mL microcentrifuge tubes, which were then set on a rotary mixer for 30 min at 4 °C followed by centrifugation at 10,000 rpm for 10 min at 4 °C. Supernatants containing soluble proteins (500 μg) were then incubated for 12 h on the rotary mixer at 4 °C with pre-washed Protein A/G Sepharose beads in 50% slurry in wash buffer (provided in the co-IP kit) and anti-LEDGF/p75 human autoantibodies (1:100 dilution). As negative control for co-IP, we used an irrelevant normal human serum (NHS) that lacked autoantibody reactivity against PCa cells, as assessed by immunoblotting and immunofluorescence microscopy. Human DFS-positive sera containing high titer, monospecific anti-LEDGF/p75 autoantibodies were used for co-IP experiments. Antigen-antibody-bead complexes were centrifuged at 2000 rpm for 2 min at 4 °C, and the bead-bound complexes were washed three times with the wash buffer. Proteins were eluted by adding 4X lithium dodecyl sulfate (LDS) buffer (Invitrogen-Thermo Fisher Scientific, Waltham, MA, USA, Cat# NP0007) containing 0.1% β-mercaptoethanol (Sigma-Aldrich, St. Louis, MO, USA, Cat# M-6250) to the beads followed by boiling for 5 min. Samples were centrifuged at 12,000 rpm for 3 min at 4 °C, and supernatants containing co-IP proteins were processed for SDS-PAGE and immunoblotting.

### 2.9. Confocal Microscopy

Cells were grown on coverslips placed inside wells of 6-well plates (100,000 cells per well) for 24 h. RPMI medium was retrieved and cells were washed with dPBS, followed by fixation with 4% formaldehyde (Electron Microscopy Sciences, Hatfield, PA, USA, Cat# 15712) and permeabilization with 0.2% Triton X-100 (Thermo Fisher Scientific, Waltham, MA, USA, Cat# BP151-100). To reduce non-specific fluorescence, cells were first incubated for 1 h in blocking buffer (12.5% BSA, 10% Triton-X100, and 0.5% Tween-20 in dPBS) and then co-incubated with human anti-LEDGF/p75 autoantibodies together with rabbit or mouse antibodies to individual interacting partners for 2 h at room temperature. All antibodies were used at 1:200 dilution. Cells were incubated with appropriate secondary antibodies labeled with FITC or rhodamine at 1:50 dilution for 1 h, and coverslips were mounted on slides with medium containing 4′,6-diamidino-2-phenylindole (DAPI; Vectashield, Burlingame, CA, USA, Cat# H-1200-10). Confocal microscopy was conducted using a Zeiss LSM-710-NLO microscope with a 63X oil immersion objective and appropriate filters. Images were analyzed using ImageJ.

### 2.10. Nuclear Detection of JPO2

For the nuclear detection of JPO2, PC3-DR, and DU145-DR, cells were treated with 100 nM dexamethasone (Sigma-Aldrich, St. Louis, MO, USA, Cat# D4902) for up to 1.5 h prior to cellular fractionation or confocal analysis. For the cellular fractionation experiments, cells were seeded in 100 mm tissue culture dishes, allowed to adhere in humidified 37 °C/5% CO_2_ incubator for 24 h, and then incubated for 24 h in RPMI medium supplemented with 10% charcoal-stripped FBS (CS-FBS; Gibco Cell Culture-Thermo Fisher Scientific, Waltham, MA, USA, Cat# 12676-029) prior to treatment with dexamethasone. Cells were treated with dexamethasone, trypsinized, washed with dPBS, and centrifuged at 1500 rpm for 5 min at 4 °C. Pellets were resuspended in 100 μL of 1X hypotonic lysis buffer supplemented with PIC and incubated on ice for 15 min. Igepal CA-630 (Sigma-Aldrich, St. Louis, MO, USA, Cat# I3021) was added (0.6%), and tubes were vortexed vigorously for 10 s and immediately centrifuged at 9500 rpm for 30 s at 4 °C. Supernatants (cytoplasmic fraction) were transferred to pre-chilled tubes for subsequent studies. Pellets containing nuclei were resuspended in 100 μL of Laemmli lysis buffer supplemented with complete PIC and 100 mM phenylmethanesulfonylfluoride (PMSF). Lysates were then sonicated and individually passed through a 50 μL 22-gauge Hamilton syringe (Hamilton Company, Reno, NV, USA, Cat# 80565) to shear DNA and reduce sample viscosity. Samples were centrifuged at 12,000 rpm for 5 min at 4 °C, and the supernatants (soluble nuclear fraction) were transferred to pre-chilled tubes for use in SDS-PAGE and immunoblotting. For the nuclear visualization of JPO2, cells were grown overnight in 6-well plates containing coverslips before incubation in CS-FBS RPMI-1640 medium for 16 h at 37 °C/5% CO_2_, followed by replacement with the same medium with or without 100 nM dexamethasone and processing for confocal microscopy.

### 2.11. RNA Interference

PC3-DR and DU145-DR cells (50,000 cells per well) were cultured on 6-well plates and transfected 24 h later with either 50 nM LEDGF/p75, 100 nM JPO2, 100 nM menin, or 200 nM HRP2 siRNAs for up to 96 h. The siRNA sequences used were as follows: si-LEDGF/p75 (′5-AGACAGCAUGAGGAAGCGAUU-3′), validated previously [9,18,42]; si-JPO2 (pool of three siRNAs, A = ′5-UGAAAGGCUACUCGAAGACUU-3′, B = ′5-UUAUCUCGAACAGUUAU GGTT-3′, C = ′5-UUAGGCACCAAUGGUAUGCTT-3′); si-menin (′5-GAUCAUGCCUGG GUAGUGUUUG-3′), selected from a pool of 10 siRNAs validated previously [43]; si-HRP2 (pool of three different siRNA duplexes from Santa Cruz Biotechnology, Dallas, TX, USA, Cat# sc-105539). Cells were transfected using Interferin^®^ siRNA transfection reagent (Polyplus-transfection^®^, Illkirch, France, Cat# 409-01). Scrambled siRNA duplex (SD, Dharmacon, Lafayette, CO, USA, Cat# D-001210-0105) was used as non-targeting negative control. Protein depletion was assessed by immunoblotting.

### 2.12. Apoptosis Assays

PC3-DR and DU145-DR cells were seeded at a density of 50,000 cells per well in six-well plates and incubated in 2 mL of RPMI medium containing 10% FBS, penicillin/streptomycin, and normocin. After 24 h, the cells were transfected with either 50 nM LEDGF/p75, 100 nM JPO2, 100 nM menin, or 200 nM HRP2 siRNAs for 72 h. Scrambled siRNA duplex (SD) was used as non-targeting negative control. Supernatants from each of the wells were collected prior to detachment of the cell monolayer with diluted trypsin (1 min treatment) and harvesting. The combined floating and attached harvested cells for each condition were used for analysis, and samples were kept on ice. Annexin V/7AAD staining was performed according to the recommended protocol of the Annexin V Apoptosis Detection Kit eFluor™ 450 (eBioscience-Thermo Fisher Scientific, Waltham, MA, USA, Cat# 88800672). Fluorescence was measured using a Miltenyi Biotec MACSQuant Analyzer 10 Flow Cytometer (Miltenyi Biotec, Auburn, CA, USA). The percentage of apoptotic cells (Annexin V positive) was determined using FlowJo software version 9.9.6 (FlowJo, Ashland, OR, USA).

### 2.13. Clonogenic Assays

PC3-DR and DU145-DR cells were transfected with individual siRNAs and grown in RPMI-1640 medium supplemented with 10% FBS for 72 h. Then, an equal number of viable transfected cells was transferred to 6-well culture plates (500 cells per well). Twenty-four hours later, new medium containing DTX was added, and plates were incubated for 10 days at 37 °C/5% CO_2_. Adherent colonies were washed with dPBS, fixed with ice-cold 3:1 (*v*/*v*) methanol–acetic acid solution for 5 min, washed again with dPBS, stained with 0.5% crystal violet for 20 min, and then washed gently with tap water. Images of the stained colonies were acquired using a 20-megapixel Cannon SX740-HS camera, and quantification was performed using the automated colony counting capability of Image J software following identical parameters for each well.

### 2.14. Tumorsphere Formation Assays

Spheroid cultures from siRNA transfected cells were maintained using complete MammoCult™ medium (Stem Cell Technologies, Vancouver, Canada, Cat# 05620) supplemented with hydrocortisone (0.48 μg/mL, Sigma-Aldrich, St. Louis, MO, USA, Cat# H0135), heparin (4 μg/mL Sigma-Aldrich, St. Louis, MO, USA, Cat# H3149), and 1% penicillin/streptomycin. PC3-DR and DU145-DR cells were seeded at 50,000 cells per well and transfected with the various siRNAs. After 48 h, an equal number of viable cells (1000 cells per well) were harvested and resuspended 50 times in MammoCult™ medium to ensure a single cell suspension. Cells were then seeded in 24-well untreated plates (Genesee, Cat# 25–102) in 0.5 mL MammoCult™ medium. Tumorspheres were grown for 4 days at 37 °C/5% CO_2_ and visualized in an Olympus IX70 microscope equipped with Phase Contrast and Hoffman Modulation Contrast, and a SPOT imaging system. Tumorsphere area was quantified from three independent images per individual treatment using Image J software.

### 2.15. Measurement of Surface CD44 Antigen

Cell culture and gene silencing procedures were performed as mentioned above. Cells were stained using a standard flow cytometry protocol for the detection of surface CD44 antigen using V450 Mouse Anti-human CD44 antibody according to the manufacturer’s instructions (BD Biosciences, Franklin Lakes, NJ, USA, Clone# G44-26, Cat# 561292). Flow cytometry analysis was performed using a MACSQuant analyzer 10 (Miltenyi Biotec, Auburn, CA, USA) and FlowJo analysis software (FlowJo, Ashland, OR, USA).

### 2.16. Statistical Analysis

Data are expressed as mean ± SEM from at least 3 independent experiments. Statistical analysis was performed with GraphPad Prism version 6 (GraphPad Software, San Diego, CA, USA). Two-sample comparisons were determined using the two-tailed Student *t*-test. For multiple comparisons, we used two-way ANOVA. *p* values < 0.05 were considered statistically significant.

## 3. Results

### 3.1. The LEDGF/p75 IBD Interactome Is Endogenously Overexpressed in DTX-Resistant PCa Cells

To evaluate the contribution of the LEDGF/p75 IBD interactome to PCa chemoresistance, we first assessed the endogenous protein expression of LEDGF/p75 and its known IBD-interacting partners JPO2, menin, MLL, IWS1, ASK1, PogZ, and Med-1 in AR-independent DTX-resistant PC3-DR and DU145-DR cell lines compared to their drug-sensitive, parental counterparts. We also included in our analysis c-MYC, an interacting partner of JPO2 [44], and HRP2, which is not considered an IBD-binding protein but shares significant structural and functional overlap with LEDGF/p75 [37]. The DTX-resistant cell lines PC3-DR and DU145-DR were developed by selection and expansion of surviving cells after consecutive treatments with increasing concentrations of DTX [6,9]. In a previous study, we showed via RNA sequencing and functional assays that these DTX-resistant cell lines upregulate a transcriptomic program associated with increased stemness [6]. These cell lines showed increased IC50 values, overexpression of MDR1 (multi-drug resistance protein 1), and enhanced clonogenic capacity compared to their drug-sensitive counterparts (Supplementary Figure S1).

**Figure 1:**
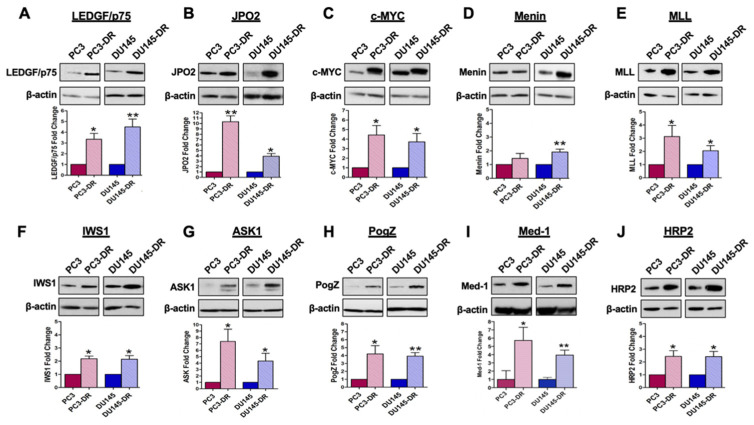
LEDGF/p75 and its IBD-interacting partners are differentially upregulated in DTX-resistant PCa cell lines. Upper panels: immunoblots showing upregulation of (**A**) LEDGF/p75, (**B**) JPO2, (**C**) c-MYC, (**D**) menin, (**E**) MLL, (**F**) IWS1, (**G**) ASK1, (**H**) PogZ, (**I**) Med-1, and (**J**) HRP2 in DTX-resistant PC3-DR and DU145-DR cells compared to their parental, drug-sensitive counterparts. Bottom panels: bar graphs showing quantification of fold change in protein expression from at least three independent experiments per cell line via densitometric ImageJ analysis, with values normalized to β-actin. Paired *t*-test statistical analysis revealed that protein expression in DTX-resistant PCa cells achieved statistical significance compared to controls (* *p*  <  0.05, ** *p*  <  0.01). Due to the very low expression of some of the proteins in the parental PC3 and DU145 cells, we normalized their expression to an arbitrary value of 0.10 for quantification purposes. Error bars represent mean ± standard deviation (SD).

Consistent with our previous observations [6,9], LEDGF/p75 was upregulated in PC3-DR and DU145-DR cells compared to their respective drug-sensitive parental cells (Figure 1A). JPO2 and c-MYC were also upregulated in both cell lines (Figure 1B,C). Menin and MLL, which form a ternary complex with both LEDGF/p75 and HRP2 through IBD binding in leukemia cells [19,37], were overexpressed in DU145-DR cells (Figure 1D,E). However, while MLL was significantly overexpressed in PC3-DR cells, menin showed moderately increased expression without achieving statistical significance (Figure 1D,E). Other LEDGF/p75 IBD-interacting proteins, IWS1, ASK1, and PogZ, were also overexpressed in the PC3-DR and DU145-DR cells compared to DTX-sensitive cells (Figure 1F–H). Med-1, recently reported as an interacting partner of the LEDGF/p75 IBD [15,39], was also significantly overexpressed in the chemoresistant cell lines compared to the parental sensitive controls (Figure 1I). In addition, HRP2 was significantly overexpressed in both DTX-resistant cell lines (Figure 1J). The fold induction values for each of these proteins in the DTX-resistant cells relative to their sensitive counterparts are listed in Table 1. The upregulation of these IBD-interacting proteins appeared to be posttranscriptional since, with the exception of LEDGF/p75 and c-MYC, we did not find in pilot qPCR studies or in RNAseq data [6] consistently increased transcript expression in the DTX-resistant cells (data not shown).

### 3.2. LEDGF/p75 Interacts Endogenously with IBD-Binding Partners in DTX-Resistant PCa Cells

After establishing the upregulation of LEDGF/p75 and members of its IBD interactome in PC3-DR and DU145-DR cells, we sought to determine if these protein interactions also occur endogenously in the chemoresistant cells. First, we conducted an ingenuity pathway analysis (IPA) to identify in silico if any protein–protein interaction data are already available on individual members of the LEDGF/p75 IBD interactome in PCa cells. IPA results showed that c-MYC was associated with 26 canonical pathways, ASK1 (MAP3K5) with 23 pathways, and MLL (KMT2A) with 1 pathway (Table S1). An IPA map of a representative canonical pathway (Molecular Mechanisms of Cancer) showing protein interactions involving c-MYC and ASK1 in PC3 cells is provided in Supplementary Figure S2. Similar results were obtained with DU145 cells (data not shown). These results indicated that while there are substantial data on protein interactions or pathways involving these two proteins in PCa cells, there are limited data for LEDGF/p75 and its interacting partners.

**Figure 2:**
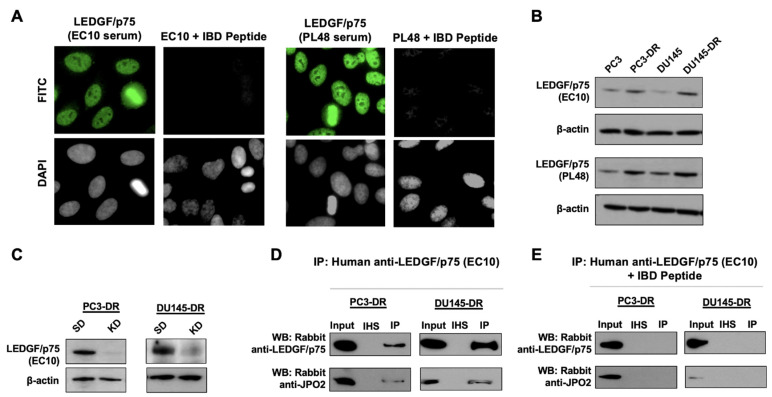
Validation of the specificity of human anti-LEDGF/p75 autoantibodies used in this study. (**A**) Representative human monospecific (only fluorescence pattern detected) anti-LEDGF/p75 sera (EC10 and PL48) displaying the characteristic dense fine speckled (DFS) nuclear pattern of high titer anti-LEDGF/p75 autoantibodies, also known as anti-DFS or anti-DFS70 autoantibodies (FITC), in HEp-2 antinuclear antibody (ANA) test slides. Pre-adsorption of the two human anti-LEDGF/p75 sera with the autoepitope-containing IBD peptide (LEDGF/p75 residues 347–429) abolished their immunoreactivity in HEp-2 ANA test slides. (**B**) Immunoblots showing immunoreactivity of the selected anti-LEDGF/p75 sera against a 75 kD protein band in whole cell lysates of both parental and DTX-resistant PC3 and DU145 cell lines, with noticeable increased expression of the protein in the resistant cells. (**C**) Immunoblots showing the reactivity of representative human serum EC10 against LEDGF/p75 in whole lysates from PC3-DR and DU145-DR cells, with siRNA-mediated LEDGF/p75 knockdown (KD) compared to scrambled duplex control (SD). Note the loss of serum immunoreactivity against the 75 kD protein band in cells with LEDGFp75 knockdown. Beta-actin was used as loading control in panels B and C. (**D**) Immunoprecipitation of endogenous LEDGF/p75 from PC3-DR and DU145-DR cells using representative anti-LEDGF/p75 human serum EC10. A well-characterized LEDGF/p75 interacting partner, JPO2, was used as a positive control to initially monitor and validate the capacity of monospecific anti-LEDGF/p75 sera to immunoprecipitate its IBD-interacting proteins. Irrelevant normal human serum (NHS) was used as negative control. (**E**) Pre-adsorption of anti-LEDGFp75 human EC10 serum with the autoepitope-containing IBD polypeptide abolished its ability to co-immunoprecipitate LEDGF/p75 and JPO2, again confirming the specificity of the serum autoantibodies.

To determine if LEDGF/p75 interacts endogenously with its known IBD-interacting partners in DTX-resistant cells, we immunoprecipitated proteins from PC3-DR and DU145-DR cells using human anti-DFS sera containing autoantibodies to LEDGF/p75 [42]. These polyclonal but highly specific human autoantibodies react with multiple epitopes within the entire IBD region [45], which allows for their immunoprecipitation of LEDGF/p75 without necessarily competing with its IBD-interacting partners for binding sites. To validate the specificity of the two anti-DFS patient sera (EC10 and PL48) used in our co-immunoprecipitation (co-IP) studies, we first pre-absorbed these sera with a recombinant peptide corresponding to the entire LEDGF/p75 IBD autoepitope region. The pre-absorbed sera lost their nuclear DFS immunofluorescence staining pattern characteristic of antibodies to LEDGF/p75 (Figure 2A). Immunoblotting analysis confirmed that both sera detected the increased LEDGF/p75 expression observed in the DTX-resistant PC3-DR and DU145-DR cells compared to sensitive cells (Figure 2B). In addition, LEDGF/p75 depletion using specific siRNAs abolished the anti-DFS serum immunoreactivity in both cell lines (Figure 2C). Finally, the ability of anti-DFS sera to co-immunoprecipitate LEDGF/p75 and its known IBD interacting partner JPO2 was abolished by pre-absorption with the IBD autoepitope-containing peptide (Figure 2D,E). These results demonstrated that the human DFS sera EC10 and PL48 contained highly specific anti-LEDGF/p75 autoantibodies capable of reacting with this protein by immunoblotting, immunofluorescence microscopy, and immunoprecipitation and, therefore, are excellent tools to study the endogenous LEDGF/p75 interactome.

**Figure 3:**
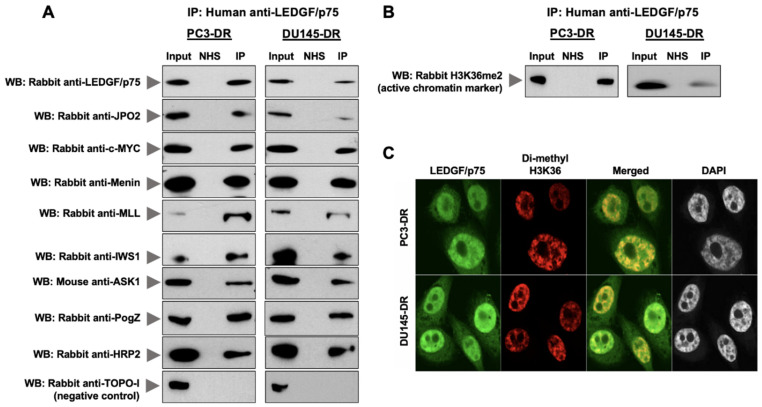
Human anti-LEDGF/p75 autoantibodies capture endogenous interactions with IBD-binding partners in DTX-resistant PCa cells. (**A**) Endogenous proteins from PC3-DR and DU145-DR cells were immunoprecipitated using highly specific and validated human sera containing autoantibodies to LEDGF/p75. Immunoprecipitated LEDGF/p75 and its endogenous IBD-interacting partners were detected by immunoblotting using specific rabbit or mouse antibodies. Proteins in cell lysates were also immunoprecipitated using an irrelevant normal human serum (NHS) as negative control. Input represents 1% of the protein concentration of the immunoprecipitated cell lysate. TOPO-1 was included as a negative control since there is no evidence of its interaction with LEDGF/p75. (**B**) Di-methyl histone H3K36 (H3K36me2), a marker for active chromatin, was immunoprecipitated with anti-LEDGF/p75 autoantibodies. (**C**) Rabbit antibody to H3K36me2 was co-incubated with human anti-LEDGF/p75 autoantibodies in DTX-resistant cells and detected with rhodamine-labeled secondary antibody (red). Merged images show the yellow-orange staining typical of colocalization. DAPI was used for nuclear staining. Images were acquired by confocal microscopy. Scale bar, 10 µm, applies to all confocal images.

LEDGF/p75 and its IBD interacting partners were co-immunoprecipitated with the human autoantibodies and detected by immunoblotting using commercially available antibodies (Figure 3A). Our results show that LEDGF/p75 co-immunoprecipitated with its endogenous IBD interacting partners JPO2, menin, MLL, IWS1, ASK, and PogZ in both PC3-DR and DU145-DR cells (Figure 3A). c-MYC and HRP2 were also co-immunoprecipitated. We were unable to detect Med-1 in the co-IPs, most likely due to the low abundance of this protein in PCa cells or relatively poor Western blot transfer due to its high molecular weight. In order to detect this protein in the immunoblots shown in Figure 1I, we had to load 7–10 times more cell lysate protein than for the other proteins examined. Endogenous proteins in cell lysates were also immunoprecipitated with an irrelevant normal human serum (NHS) used as a negative control. Human autoantibodies specific to TOPO-1/Scl-70, a protein that has not been implicated as a LEDGF/p75 binding partner, were also used as negative control to confirm the specificity of LEDGF/p75 protein–protein interactions. As expected, TOPO-1 was not co-immunoprecipitated by the anti-LEDGF/p75 autoantibodies, and NHS did not immunoprecipitate any of the proteins examined. To determine the co-IP efficiency, we compared by immunoblotting analysis the amount of immunoprecipitated LEDGF/p75 from PC3-DR and DU145-DR whole cell lysates (eluate) with that of input (1% of eluted fraction), irrelevant NHS, and flow-through (unbound material) (Supplementary Figure S3). These results highlighted the specificity of the autoantibody-mediated co-IP of the LEDGF/p75 interactome.

H3K36me2, a marker of active chromatin that is recognized by the LEDGF/p75 PWWP domain [25], also co-immunoprecipitated with LEDGF/p75 (Figure 3B). Confocal microscopy analysis of endogenous H3K36me2 and LEDGF/p75 showed a noticeable co-localization, most likely in areas of the nucleus corresponding to active chromatin (Figure 3C). This suggested that the LEDGF/p75 endogenous interactome is located within active chromatin in the chemoresistant cells.

### 3.3. LEDGF/p75 Colocalizes with Interacting Partners in Nuclei of DTX-Resistant PCa Cells

For subsequent studies, we selected the LEDGF/p75 IBD interactome members JPO2, c-MYC, menin, and MLL, which while previously implicated in PCa [40,41,44] have not been investigated together with LEDGF/p75 in the context of PCa chemoresistance. We also included in this analysis the LEDGF/p75 paralog HRP2. Using confocal microscopy, we investigated if LEDGF/p75 co-localizes endogenously in the nucleus of DTX-resistant cells with these selected members of its interactome.

**Figure 4:**
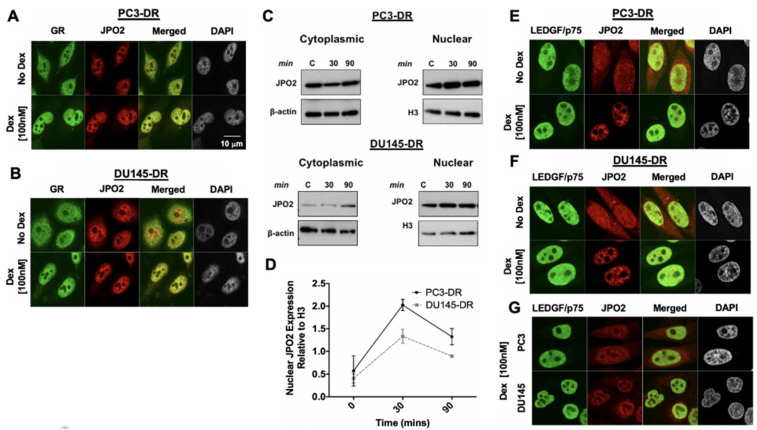
JPO2 co-localizes with LEDGF/p75 upon dexamethasone-induced nuclear translocation. (**A**,**B**) Dexamethasone induced JPO2 nuclear translocation in the DTX-resistant PCa cells PC3-DR and DU145-DR. Cells growing in charcoal-stripped medium were treated with 100 nM dexamethasone (Dex) for 30 min and then prepared for confocal microscopy analysis using an anti-JPO2 mouse antibody co-incubated with an anti-GR rabbit antibody used as a control for nuclear translocation. (**C**) Cells growing in charcoal-stripped medium were treated with 100 nM Dex for up to 90 min, followed by cytoplasmic and nuclear protein extraction for immunoblotting detection of GR and JPO2. (**D**) Immunoblot quantification of nuclear JPO2 levels (2 independent experiments) following exposure of PC3-DR and DU145-DR cells to Dex, using ImageJ software. (**E**–**G**) Representative human anti-LEDGF/p75 antibody displays the characteristic dense fine speckles (DFS) nuclear pattern, detected with FITC-labeled secondary anti-human antibody (green). This pattern was also produced by anti-JPO2 after Dex treatment in PC3-DR (**E**) and DU145-DR (**F**) cells and their sensitive counterparts (**G**). Scale bar, 10 µm, applies to all confocal images.

In initial studies, we noticed a diminished nuclear co-localization of LEDGF/p75 and JPO2 in PC3-DR and DU145-DR cells given the elevated cytoplasmic localization of JPO2. Since JPO2 function is linked to glucocorticoid signaling [46], we treated PC3-DR and DU145-DR cells with 100 nM dexamethasone, which led to increased nuclear accumulation of JPO2 in both cell lines (Figure 4A,B) compared to untreated cells. As a positive control, we followed the nuclear translocation of glucocorticoid receptor (GR) upon dexamethasone treatment in both cell lines and noticed its nuclear co-localization with JPO2, suggesting their presence in the same transcription complex (Figure 4A,B). Glucocorticoid-induced JPO2 nuclear translocation was confirmed by immunoblotting of cytoplasmic and nuclear fractions from DTX-resistant cells treated with dexamethasone (Figure 4C,D). Next, we analyzed the co-localization of LEDGF/p75 with JPO2 in DTX-resistant cells with and without dexamethasone treatment. In the absence of dexamethasone, JPO2 appeared both in the cytoplasm and the nucleus of the DTX-resistant cells (Figure 4E,F upper panels) but localized mostly to the nucleus after dexamethasone treatment (Figure 4E,F lower panels). Translocated JPO2 partially co-localized with LEDGF/p75, likely in areas of the nucleus corresponding to active chromatin, as evidenced by the similarity between the LEDGF/p75-JPO2 co-localization pattern (Figure 4C,D) and that of LEDGF/p75-H3K36me2 (Figure 3C). LEDGF/p75 and JPO2 also co-localized in parental, drug-sensitive PC3 and DU145 cells, but the signals were weak due to their relatively lower expression in these cell lines compared to DTX-resistant cells.

**Figure 5:**
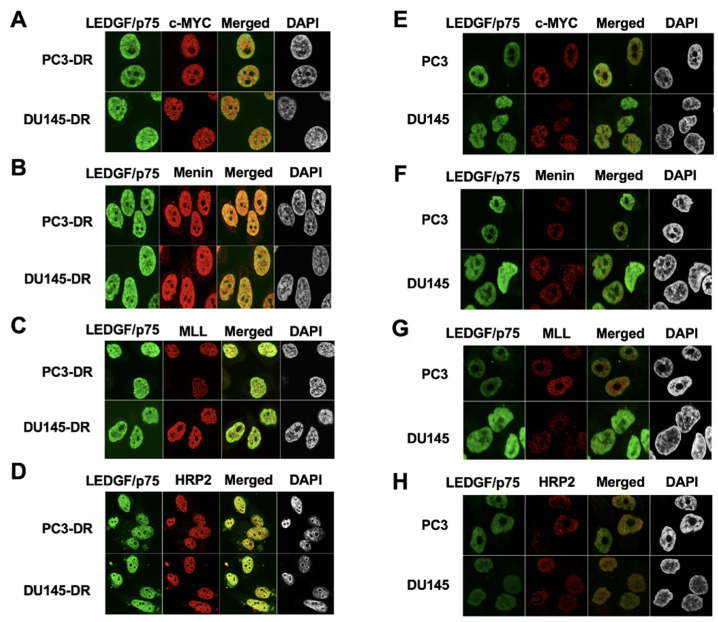
LEDGF/p75 co-localizes with selected IBD-interacting partners in DTX-resistant PCa cells. Rabbit antibodies to c-MYC (**A**,**E**), menin (**B**,**F**), MLL (**C**,**G**), and HRP2 (**D**,**H**) were co-incubated with human anti-LEDGF/p75 auto-antibodies in DTX-resistant and DTX-sensitive cells, and detected with rhodamine-labeled secondary antibody (red). Merged images show the yellow-orange staining typical of co-localization. DAPI was used for nuclear staining. Images were acquired by confocal microscopy.

Consistent with the co-IP data, LEDGF/p75 also partially co-localized in the nuclei of PC3-DR and DU145-DR cells with c-MYC, menin, MLL, and HRP2 (Figure 5A–D). However, the co-localization of these proteins in the parental DTX-sensitive cells PC3 and DU145 was scarcely detectable due to their relatively low expression compared to DR cells (Figure 5E–H).

### 3.4. LEDGF/p75 Depletion Does Not Alter the Protein Expression of Its Interacting Partners but May Influence Their Nuclear Localization

**Figure 6:**
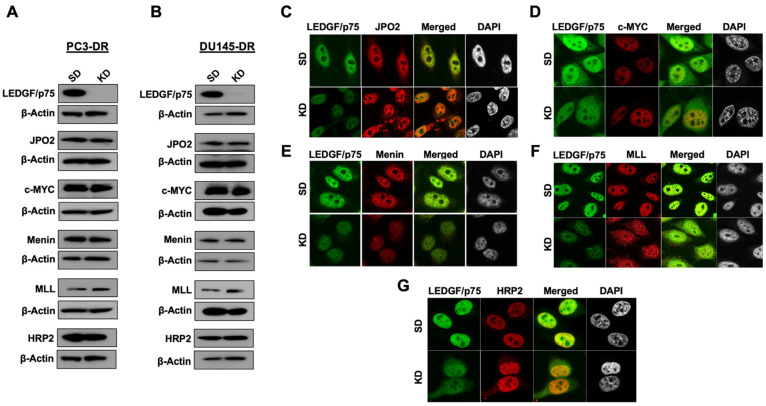
LEDGF/p75 depletion does not alter the protein expression of selected IBD interacting partners in DTX-resistant cells but affects their nuclear localization patterns. LEDGF/p75 knockdown was assessed by immunoblotting using a rabbit anti-LEDGF/p75 antibody in PC3-DR (**A**) and DU145-DR (**B**) cells transfected with siLEDGF/p75 (KD) or scrambled duplex (SD) control oligos. Representative blots show that the protein expression levels of JPO2, c-MYC, menin, MLL, and HRP2 were not altered by LEDGF/p75 depletion. For confocal microscopy analysis, human anti-LEDGF/p75 autoantibodies were co-incubated with rabbit antibodies to JPO2 (**C**), c-MYC (**D**), menin (**E**), MLL (**F**), and HRP2 (**G**) in DTX-resistant PC3 cells with and without LEDGF/p75 depletion. Detection of nuclear proteins was achieved with FITC-labeled secondary anti-human antibody (green) and rhodamine-labeled secondary anti-rabbit antibody (red). Merged images show the yellow-orange staining typical of co-localization. DAPI was used for nuclear staining.

We showed previously that ectopic LEDGF/p75 overexpression in PC3 cells confers resistance to DTX-induced cell death, whereas its depletion in PC3-DR and DU145-DR resensitizes these cells to taxanes [8,9]. This depletion likely results in decreased IBD protein–protein interactions, leading to decreased transcription of stress survival genes and attenuated cell survival. Hence, we evaluated the effects of transient LEDGF/p75 knockdown on the expression and co-localization of selected interacting partners in DTX-resistant cells. LEDGF/p75 silencing did not affect the endogenous protein expression of JPO2, c-MYC, menin, MLL, and HRP2, assessed by immunoblotting in PC3-DR (Figure 6A) and DU145-DR cells (Figure 6B).

We then visualized by confocal microscopy the effects of LEDGF/p75 depletion on the nuclear localization of JPO2, c-MYC, menin, MLL, and HRP2 in PC3-DR cells. LEDGF/p75 depletion did not affect extensively the nuclear localization of JPO2; however, there was increased punctuated cytoplasmic JPO2 staining in the knockdown cells compared to control cells (Figure 6C), suggesting that LEDGF/p75 may partially contribute to JPO2 nuclear localization. The nuclear localization of c-MYC, a JPO2-binding protein, was not affected by LEDGF/p75 depletion (Figure 6D). Interestingly, the menin signal intensity consistently decreased in the nuclei of LEDGF/p75-depleted cells, even when the total protein levels were not affected by LEDGF/p75 depletion (compare Figure 6A,B with Figure 6E, bottom panels). MLL, which is tethered together with menin to active chromatin sites by LEDGF/p75 in leukemic cells [19], displayed an altered immunofluorescence pattern in the LEDGF/p75-depleted DR cells characterized by discrete small speckles disseminated through both the nucleus and cytoplasm (Figure 6F, bottom panels). This pattern differed from that observed in the control DR cells, where LEDGF/p75 and MLL markedly co-localized in the nucleus, excluding the nucleoli (Figure 6F, top panels). These results suggested that LEDGF/p75 contributes to the endogenous nuclear localization of the menin–MLL complex. Similar to c-MYC, the endogenous nuclear localization of HRP2 was not affected by LEDGF/p75 depletion (Figure 6G).

### 3.5. Depletion of LEDGF/p75, JPO2, Menin, or HRP2 Enhances Apoptosis in DTX-Resistant Cells

**Figure 7:**
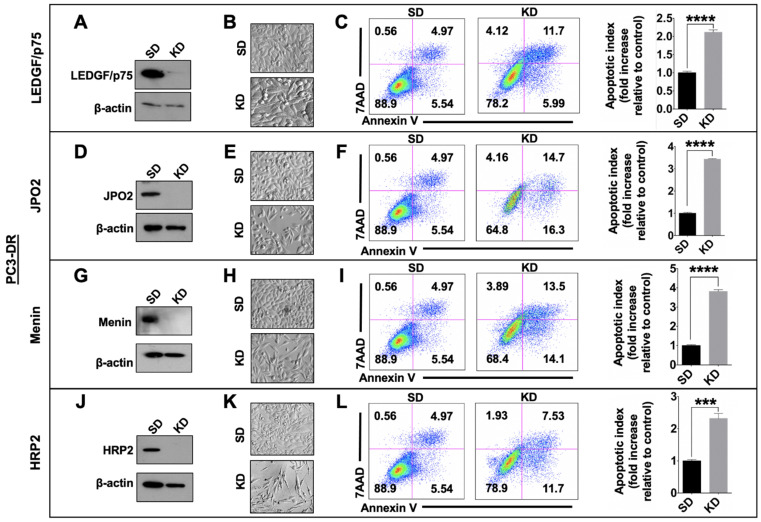
Transient knockdown of LEDGF/p75, JPO2, menin, or HRP2 decreases the survival of PC3-DR cells. siRNA-mediated silencing of LEDGF/p75 (**A**), JPO2 (**D**), menin (**G**), and HRP2 (**J**) was confirmed by immunoblotting of PC3-DR cell lysates transfected with specific siRNAs (KD) or si-SD control oligos for 72 h. PC3-DR cell morphology was visualized by Hofmann Modulation Contrast microscopy (**B**,**E**,**H**,**K**). PC3-DR cells transfected with either control (SD) or specific siRNAs (KD) were stained with Annexin V/7AAD, and cell death was analyzed by flow cytometry (**C**,**F**,**I**,**L**). Representative plots from three replicate experiments showing apoptotic index (calculated as described in Materials and Methods) are presented. Statistical significance was determined by comparing the fold increase in apoptotic cells from cells with LEDGF/p75, JPO2, menin, or HRP2 knockdown with the normalized values for cells transfected with SD control oligos, using Student’s *t*-test (*** *p* < 0.001, **** *p* < 0.0001).

To determine if individual silencing of LEDGF/p75, JPO2, menin, or HRP2 induced apoptotic cell death, we performed an Annexin V/7AAD apoptosis assay in PC3-DR and DU145-DR cells transfected with siRNAs targeting these proteins. Protein knockdowns were monitored by immunoblotting for up to 96 h, and their effects on cell morphology and survival were assessed. The optimal depletion time for all proteins was 72–96 h using optimized siRNAs (individual or pools) transfected into cells with the Interferin^TM^ reagent (see Materials and Methods). This produced robust and durable knockdowns (KD) in both DTX-resistant cell lines, as measured by immunoblotting and qPCR (Figure 7A,D,G,J, Supplementary Figure S4A,D,G,J, and Supplementary Figure S5). In a representative experiment, PC3-DR cells transfected with non-targeting control oligos (SD) showed 88.9% cell viability (Annexin V-, 7AAD-), 5.54% apoptosis (Annexin V+, 7AAD-), 4.97% late apoptosis/secondary necrosis (Annexin V+, 7AAD+), and 0.56% necrosis (Annexin V-, PI+) (Figure 7C). By contrast, LEDGF/p75 silencing in these cells caused an increase in cell death (5.99% early apoptosis + 11.7% late apoptosis + 4.12% necrosis). This silencing generated a significant fold increase in the average apoptotic index (early + late apoptotic cells) of 2.11 relative to SD control (Figure 7C). Silencing of JPO2, menin, and HRP2 also produced significant fold increases in apoptotic index averaging 3.43, 3.82, and 2.32, respectively (Figure 7F,I,L). Likewise, the silencing of LEDGF/p75, JPO2, menin, and HRP2 in DU145-DR cells led to significant fold increases in the apoptotic index, with average values of 1.48, 1.71, 2.08, and 2.32, respectively (Supplementary Figure S4). These results indicated that these four members of the LEDGF/p75 interactome contribute to the survival of PC3-DR and DU145-DR cells and were in harmony with the increased cell death visualized by morphological analysis of the cells under Hoffman Modulation Contrast microscopy (Figure 7 and Supplementary Figure S4, panels B,E,H,K).

### 3.6. Depletion of LEDGF/p75, JPO2, Menin, or HRP2 Inhibits the Clonogenicity and Tumorsphere Formation Capacity of DTX-Resistant PCa Cells

**Figure 8:**
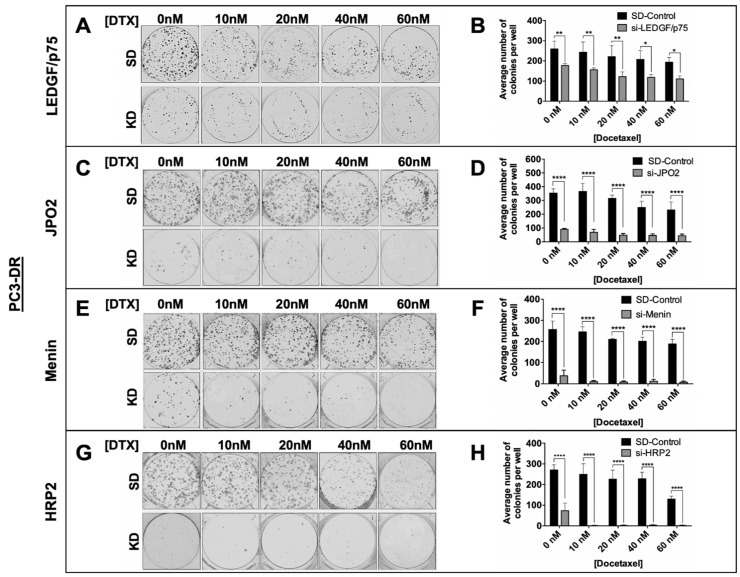
Knockdown of LEDGF/p75, JPO2, menin, or HRP2 inhibits the clonogenicity of PC3-DR cells. Representative images of clonogenic assay plates show decrease in colony formation in PC3-DR cells with LEDGF/p75 (**A**), JPO2 (**C**), menin (**E**), or HRP2 (**G**) knockdowns (KD) compared to scrambled duplex control cells (SD) in the presence and absence of DTX. Colonies were counted after 10 days of treatment. Adjacent bar graphs show quantification of PC3-DR colonies (**B**,**D**,**F**,**H**) and represent the average of colonies counted in at least three independent experiments. SEM was calculated. Statistical significance was determined by comparing the values for cells transfected with si-SD control oligos with values for cells with LEDGF/p75, JPO2, menin, or HRP2 knockdown in the presence or absence of DTX, using Student’s *t*-test (* *p* < 0.05, ** *p* < 0.01, **** *p* < 0.0001).

The upregulation of the LEDGF/p75 IBD interactome in DTX-resistant PCa cells and its impact on cell survival led us to investigate the effects of individual silencing of LEDGF/p75, JPO2, and menin on the clonogenicity and tumorsphere formation capacity of PC3-DR and DU145-DR cells. Again, because of its structural and functional similarity to LEDGF/p75, HRP2 was included in these experiments. We used LEDGF/p75 for comparison purposes in these experiments given that we previously reported that its depletion in PC3-DR and DU145-DR cells leads to a significant decrease in clonogenicity in the presence of increasing concentrations of DTX [9]. Consistent with our previous observations, LEDGF/p75 depletion led to significant reduction of colony formation in both PC3-DR (Figure 8A,B) and DU145-DR (Supplementary Figure S6A,B) cells. The clonogenicity of both control and LEDGF/p75-depleted cells gradually decreased as the resistant cells were exposed to increasing concentrations of DTX, with more robust effects produced by the combination of knockdown plus DTX. Individual depletion of JPO2, menin, or HRP2 also led to significant reduction of colony formation in PC3-DR cells (Figure 8C–H). This reduction was more robust than that observed with LEDGF/p75 depletion, particularly in the presence of DTX. Notably, knockdown of menin or HRP2 nearly abolished colony formation in the presence of DTX (Figure 8E–H). Similar results were obtained with DU145-DR cells (Supplementary Figure S6C–H), although the effects of JPO2 knockdown were not as robust as those observed in PC3-DR cells (Supplementary Figure S6C,D). We did not include parental sensitive cells in this analysis because they display lower clonogenic capacity compared to DR cells (Supplementary Figure S1) and do not form clones in the presence of DTX (data not shown).

**Figure 9:**
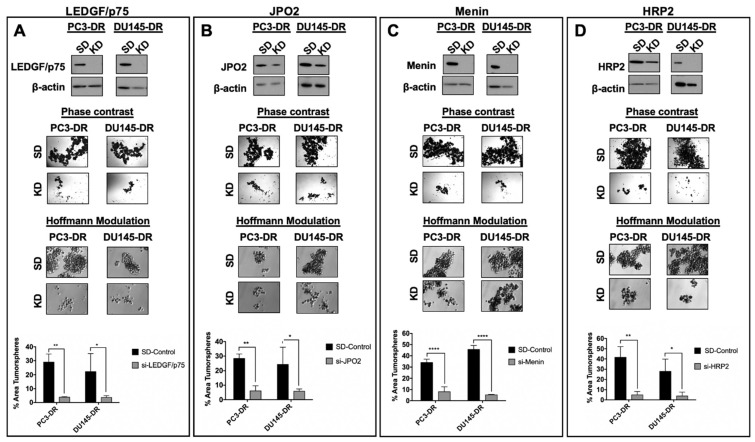
Knockdown of LEDGF/p75, JPO2, menin, or HRP2 impairs tumorsphere formation in PC3-DR and DU145-DR cells. siRNA-mediated knockdowns of LEDGF/p75 (**A**), JPO2 (**B**), menin (**C**), and HRP2 (**D**) were confirmed by immunoblotting in PC3-DR and DU145-DR cells transfected with specific siRNAs (KD) compared to cells transfected with SD control oligos. Representative images of tumorsphere formation assays showing a decrease in spheroid size in PC3-DR and DU145-DR cells after individual siRNA-mediated depletion of LEDGF/p75, JPO2, menin, or HRP2 compared to SD control spheres. Tumorspheres were visualized using phase contrast or Hoffman Modulation Contrast microscopy. Tumorsphere area was quantified (bottom graphs) from triplicate images per experiment using ImageJ software. Data are from at least 3 independent experiments and are represented as mean SEM. * *p* < 0.05, ** *p* < 0.01, **** *p* < 0.0001.

In addition, we examined the contribution of LEDGF/p75, JPO2, menin, and HRP2 to tumorsphere formation in PC3-DR and DU145-DR cells. We have shown previously that unlike the parental, drug-sensitive cells, these DTX-resistant cell lines display enhanced tumorsphere formation capacity associated with increased stemness compared to their DTX-sensitive counterparts [6], consistent with emerging evidence that tumor spheroids are enriched for CSC-like cells [47]. For these experiments, DTX-resistant cells were transiently transfected with siRNAs targeting LEDGF/p75 (Figure 9A top panel), JPO2 (Figure 9B top panel), menin (Figure 9C top panel), or HRP2 (Figure 9D top panel) for 48 h and then cultured as 3D spheroids for 5 days. The tumorsphere formation capacity of both PC3-DR and DU145-DR cells was markedly impaired when each of the selected members of the LEDGF/p75 interactome was individually silenced (Figure 9). Taken together, these results demonstrated for the first time that selected members of the LEDGF/p75 interactome contribute to the clonogenic and tumorsphere capacity of DTX-resistant PC3 and DU145 cells.

### 3.7. Depletion of LEDGF/p75 in PCa Cells Does Not Lead to Decrease in Lineage-Specific or Stemness Markers

While LEDGF/p75 has been implicated in stem cell renewal in leukemia [15], it is not clear if this protein is essential for maintaining cell lineage-specific or stemness markers in PCa cells. We first examined the effects of LEDGF/p75 silencing on the expression of GR, a nuclear steroid receptor that is upregulated in DTX-resistant PCa cells [48], and observed no impact on its protein expression levels in PC3-DR and DU145-DR cells (Supplementary Figure S7A,B). Similarly, we observed that LEDGF/p75 depletion had no effects on the protein expression levels of the nuclear receptors AR and ARv7 in the androgen-dependent cell line 22Rv1 (Supplementary Figure S7C). These results suggested that the expression of the nuclear steroid receptors AR and GR, which drive PCa progression [3,4], is not regulated by LEDGF/p75.

Previously, we showed that the PC3-DR and DU145-DR cell lines have increased stemness markers compared to the sensitive parental cells [6]. To determine if LEDGF/p75 contributes to this stemness, we evaluated the effects of its silencing on the expression of the stem cell marker Oct-4 in PC3-DR and DU145-DR cells, observing no effects (Supplementary Figure S8A). Further, LEDGF/p75 silencing had no effect on the surface expression of the cancer stem cell marker CD44 in PC3-DR and DU145-DR cells (Supplementary Figure S8B). Similarly, menin depletion had negligible effects on CD44 expression in both cell lines (Supplementary Figure S8C). By contrast, depletion of JPO2 and HRP2 produced a moderate albeit significant reduction in CD44 expression (Supplementary Figure S8D,E). It is not clear, however, is this reduction is linked to the robust cell death induced by the individual depletion of these proteins. These results are consistent with the observation that LEDGF/p75 depletion had no effects on the protein expression levels or nuclear localization of c-MYC (Figure 6), an emerging cancer stemness marker.

## 4. Discussion

PCa resistance to taxanes involves the interplay between multiple molecular mechanisms, including increased activation of anti-apoptotic proteins, multi-drug resistant transporters, cytokines and chemokines, stress and antioxidant proteins, AR variants, GR signaling, microtubule alterations, miRNAs, and EMT/CSC-associated signaling pathways [3,4,6,47,48,49]. Dissecting these mechanisms is essential for identifying molecular pathways or complexes that could be targeted in combination with taxanes for curative mCRPC treatments. The goals of this study were to characterize the endogenous LEDGF/p75 IBD interactome in taxane-resistant mCRPC cellular models and explore its potential as a therapeutic target to overcome this resistance.

Our group demonstrated previously that ectopic overexpression of LEDGF/p75 in PCa cells confers protection to DTX-induced cell death, its endogenous levels of LEDGF/p75 are upregulated in mCRPC cellular models selected for DTX resistance, and targeting this protein in chemoresistant PCa cells can resensitize them to taxanes [6,9]. In addition, we established that the interaction between the LEDGF/p75 N-terminal PWWP domain and MeCp2 increases the promoter activity of HSP27 [27], a LEDGF/p75 target gene implicated in PCa chemoresistance [50]. LEDGF/p75 is a druggable target, and disruption of its IBD-mediated protein interactions has been proposed as a potential therapeutic strategy for HIV/AIDS and leukemia [51,52,53]. However, while some LEDGF/p75 IBD-interacting partners have been implicated in cancer, including leukemia, medulloblastoma, and PCa [15,19,33,34,37,40,44,53,54,55,56], very little is known about their collective contribution to PCa chemoresistance. We hypothesized that this interactome is endogenously upregulated in cellular models of PCa chemoresistance and that targeting it could provide a rationale for exploring its potential as a novel therapeutic strategy to overcome taxane resistance.

To our knowledge, this is the first study where the currently known LEDGF/p75 IBD interactome is evaluated endogenously, in a specific cancer model, and in the context of cancer chemoresistance. Previous studies from our group and others have used *E. coli*-purified recombinant proteins and ectopic overexpression of tagged-recombinant proteins in common laboratory cell lines (e.g., HeLa, 293T, PC3, etc.) to establish interactions between the LEDGF/p75 PWWP and IBD domains and other transcription factors [19,27,34,35,36,37,54,57,58]. These studies have generated a wealth of valuable information on the detailed molecular interactions between these proteins and their implications for malignancy. However, studies on endogenous LEDGF/p75 protein interactions in clinically relevant cancer cellular models have been more challenging due to the relatively low expression of some IBD interactome proteins in many established cell lines, as well as the limited availability of antibodies that can efficiently co-IP endogenous LEDGF/p75 with its interacting partners. Using co-IP and confocal microscopy approaches, we took advantage of the upregulation of this interactome in DTX-resistant cells, and the availability of highly specific human anti-LEDGF/p75 autoantibodies (known as DFS or anti-DFS70 autoantibodies), to establish that LEDGF/p75 interacts endogenously with its known IBD-binding partners in a pre-clinical cellular model of cancer chemoresistance. We should note that the clinical and biological significance of these DFS autoantibodies is still unclear given their presence in subsets of healthy individuals, particularly young women and children, patients with diverse inflammatory conditions, and even subsets of patients with PCa [16,20]. They are considered as negative biomarkers of systemic autoimmune rheumatic disease and sensors of an aberrantly regulated LEDGF/p75 [16,20]. Their recognition of conformational and linear epitopes along the entire IBD region [45] is still puzzling but makes them valuable tools in studies of LEDGF/p75 biology.

The upregulation of the LEDGF/p75 IBD interactome in DTX-resistant PCa cells is likely to increase global RNAPII-mediated transcription, consistent with the notion that cancer cells become “addicted” to high levels of transcription to maintain their malignant phenotype, including resistance to stressors such as chemotherapeutic drugs and inhibitors of transcription [59]. It is not clear, however, what is driving the expression of this interactome in DTX-resistant PCa cells. It is well established that AR signaling drives PCa progression by upregulating the expression of cell survival and cancer-related genes in AR-positive PCa cells (2-4). However, GR bypasses AR signaling and drives the expression of both GR-target and AR-target genes, including therapy resistance genes, in PCa cells that are androgen refractory or treated with long-term anti-androgen therapy [60]. We reported previously that both AR and GR signaling upregulate LEDGF/p75 expression in metastatic PCa cells [61]. Further, as shown in Figure 4, the translocation of JPO2 into the nucleus is dependent on GR activation by glucocorticoids. Therefore, follow-up studies should determine if the expression, nuclear localization, and functions of LEDGF/p75 and its IBD interacting partners are dependent on GR signaling in GR-positive DTX-resistant cells.

Of note, c-MYC, a JPO2-interacting partner and amplifier of RNAPII-transcribed genes [44,59], was detected as part of the endogenous LEDGF/p75 interactome, although there is no evidence that the two proteins physically interact. It is likely that c-MYC associates with this interactome through its interaction with JPO2 and not through direct binding to LEDGF/p75. JPO2 and LEDGF/p75 were reported as coordinately upregulated in murine and human metastatic medulloblastoma and interact in medulloblastoma cell lines to promote P13K/AKT signaling and cell migration [54]. Recently, it was also reported that JPO2 expression is elevated in PCa and promotes disease aggressiveness by stabilizing c-MYC [44]. Thus, it is plausible that JPO2 brings c-MYC into close proximity to LEDGF/p75 to facilitate its localization to active chromatin as part of the RNAPII transcription complex. However, LEDGF/p75 alone may not be essential for this since its depletion did not affect c-MYC nuclear localization in the DTX-resistant cells.

HRP2, which in concert with LEDGF/p75 tethers IBD-interacting partners to active chromatin and facilitates RNAPII transcription [35,36,37,38], may likely facilitate the localization of c-MYC and other transcription factors to active chromatin in the absence of LEDGF/p75. Our observation that LEDGF/p75 silencing did not completely abrogate cell survival and clonogenicity in the DTX-resistant cells points to the presence of other factors that may complement its pro-survival activity (Figure 7, Figure 8, Figures S4 and S6). HRP2, for instance, interacts with other IBD interactome members (e.g., menin, MLL, and IWS1) and contributes to HIV-1 integration in LEDGF/p75-knockout cells through IBD-mediated interaction with the HIV-integrase, suggesting that in the absence of LEDGF/p75, it may still facilitate RNAPII transcription of cell survival genes [34,35]. Our observation that knockdown of HRP2, as well as JPO2, robustly abrogated the survival, clonogenicity, and tumorsphere formation capacity of DTX-resistant cells implicates for the first time these proteins in the survival of chemoresistant PCa cells. These results are consistent with recent studies showing that depletion of LEDGF/p75 sensitizes mixed-lineage leukemic cells to chemoresistance and that individual or combined silencing of LEDGF/p75 and HRP2 reduces the in vivo growth of treatment-resistant glioma [15,62].

While LEDGF/p75 silencing moderately affected the nuclear localization of JPO2, menin, and MLL, it had no effects on HRP2 and c-MYC, which would be consistent with a lack of direct physical interaction between LEDGF/p75 and HRP2 or c-MYC. However, given that LEDGF/p75 and HRP2 share significant structural and functional homology [35,36,37,38], we expected to detect both proteins in the same transcription factor complex in DTX-resistant cells. This would allow them to function together, or play redundant roles, in tethering transcription factors to active chromatin sites to promote increased RNAPII transcription of cell survival genes in the taxane-resistant cells. Consistent with this, both LEDGF/p75 and HRP2 interact with menin and MLL in mixed lineage leukemia to promote transcription of cancer-related genes, cell proliferation, and survival [19,37]. Interestingly, it was noted that the interaction of HRP2 with MLL was less dependent on menin, a protein that is critical to facilitate the interaction between LEDGF/p75 and MLL [37]. LEDGF/p75 was also recently shown to support MLL leukemia resistance to cytarabine chemotherapy, likely via interaction with co-activators of super-enhancers (SE), highlighting the role of this protein and its interactome in promoting cancer chemoresistance [15].

Emerging evidence indicates that the menin–MLL complex is upregulated in PCa, plays a role in AR signaling in AR-positive tumors, and can be targeted with small molecule inhibitors to reduce xenograft tumor growth [40]. However, its role in AR-negative, DTX-resistant cells had not been previously investigated. Our results showed for the first time that menin and MLL are upregulated in DTX-resistant, AR-independent PCa cells and interact endogenously with LEDGF/p75, and that menin knockdown inhibits the survival, clonogenicity, and tumorsphere formation of these cells. These results, combined with those obtained with JPO2 and HRP2, underline a critical role for the LEDGF/p75 IBD interactome in taxane resistance. While LEDGF/p75 silencing did not affect menin and MLL expression in DTX-resistant cells, it influenced their nuclear localization pattern. This is consistent with the role of LEDGF/p75 in tethering these and other IBD-interacting partners to active chromatin sites [33,34]. It would be important, however, to determine in future studies whether HRP2 can compensate for the absence of LEDGF/p75 in DTX-resistant cells by efficiently tethering menin, MLL and other IBD-interacting transcription factors to active chromatin.

Other members of the LEDGF/p75 interactome that were upregulated in DTX-resistant PCa cells were IWS1, ASK1, PogZ, and Med-1. While we did not investigate their contribution to DTX-resistance in PCa cells in this study, there is growing evidence supporting their role in cancer. For instance, IWS1 phosphorylation by AKT in lung cancer was shown to be important for the regulation of RNA processing [55]. ASK1 has been implicated in several cancer signaling pathways, including cellular responses to DTX therapy [56]. While the role of PogZ in cancer is poorly understood, this protein was reported to interact with HRP2 in cancer cells to facilitate DNA repair [29,63]. Med-1, a mediator of RNAPII transcription and interactor of the bromodomain protein 4 (BRD4), an SE assembly protein, was recently implicated in leukemia chemoresistance via interaction with LEDGF/p75 [15] and in enhancing the stemness and metastasis of squamous cell carcinoma via SE formation [64].

A limitation of our study is the lack of transcriptomic or proteomic data from patient-derived DTX-resistant PCa samples confirming the upregulation of members of the LEDGF/p75 interactome. It should be emphasized that these clinical samples are extremely difficult to obtain given the complexity of biopsying bone metastasis, where the DTX-resistant tumors typically reside. We searched in silico for PCa gene/protein expression datasets derived from DTX-resistant tumors but were unable to find datasets that specifically compared transcript or protein expression in taxane-naive vs. taxane-resistant prostate tumors. In light of this, future translational studies could focus on determining whether the LEDGF/p75 IBD interactome is upregulated at the transcript or protein level in circulating tumor cells or circulating exosomes from PCa patients that failed taxane therapy compared to chemotherapy-naive patients. We should note, however, that c-MYC, a member of the LEDGF/p75 interactome, has already been implicated as a driver of tumor aggressiveness, stemness, and chemoresistance in various cancer types, including PCa [41,65,66,67,68].

## 5. Conclusions

Our results revealed the following novel observations: (1) LEDGF/p75 and its IBD interacting partners are spontaneously upregulated in AR-independent PCa cells that were selected for taxane resistance; (2) using human anti-LEDGFp75 autoantibodies as immunoprecipitation tools, we showed that these proteins interact endogenously in DTX-resistant PCa cells as part of a complex that co-localizes with the active chromatin marker H3K36me2; (3) c-MYC and HRP2, two proteins that have been linked to LEDGF/p75 function, are members of this interactome in DTX-resistant PCa cells, although it is not clear if the three proteins interact directly; (4) LEDGF/p75 silencing does not affect the expression of its interacting partners but influences the nuclear localization of JPO2, menin, and MLL in DTX-resistant PCa cells; (5) individual silencing of LEDGF/p75, JPO2, menin, and HRP2 increases apoptosis and abrogates clonogenic and tumorsphere formation capacity in DTX-resistant PCa cells; and (6) silencing LEDGF/p75 does not affect the expression of lineage markers (AR or GR) or stemness associated proteins (Oct-4 and CD44) in PCa cells, suggesting that this protein may not contribute to lineage-specific nuclear steroid receptors and stemness in PCa. Although the contribution of LEDGF/p75 to chemoresistance has been documented in various cancers, our results implicate for the first time its IBD interactome in PCa chemoresistance (see visual abstract). Further studies are necessary to dissect in detail the mechanistic contribution of individual members of this interactome to DTX resistance. Although drug resistance mechanisms may differ from one cancer type to another, the recent report that LEDGF/p75-induced chemoresistance in mixed-lineage leukemia cells involves the activation of cell cycle genes, regulation of the expression of BRD4 and Med-1, and activation of nuclear SE [15] raises the question of whether LEDGF/p75 and its IBD-interacting partners, and possibly PWWP-interacting proteins, induce taxane resistance in PCa cells via similar mechanisms. Future studies must include RNAseq and ChIPseq studies, followed by functional analyses, focused on LEDGF/p75, HRP2, and other members of the interactome in the context of PCa taxane resistance to elucidate the underlying mechanisms. Additional studies targeting LEDGF/p75 and its interacting partners with small molecule inhibitors, individually and in combination, in both cell-derived and patient-derived xenograft models of DTX resistance, are guaranteed to determine the therapeutic potential of targeting this interactome for overcoming PCa chemoresistance in the clinic.

## Figures and Tables

**Table 1:** Nomenclature, functions, and average fold induction of LEDGF/p75 interactome proteins in docetaxel-resistant PC3-DR and DU145-DR cells relative to their sensitive counterparts.

Protein	Approximate MW	Other Common Names	Functions	PC3-DR *	DU145-DR *
LEDGF/p75	70 kD	DFS70, PSIP1	Stress survival, transcription coactivator of RNAPII, chromatin binding	3.35	4.50
JPO2	52 kD	CDCA7L, R1	PI3K regulator, c-MYC binding protein and potentiator	10.32	3.92
c-MYC	50 kD		Oncogenic transcription factor; cancer stemness marker	4.44	3.71
Menin	83 kD	MEN1	Histone methyltransferase, transcription factor, hematopoiesis, leukemogenesis	1.45	1.90
MLL	432 kD	KMT2A	Transcription factor, early development, hematopoiesis, leukemogenesis	3.13	2.05
IWS1	56 kD		RNAPII elongation, transcription regulator	2.18	2.15
ASK1	70 kD	MAP3K5	Stress-activated cell cycle regulating mitogen activated kinase	7.40	4.35
PogZ	155 kD		Pogo transposable element, mitosis, chromatin remodeling	4.21	3.93
Med-1	168 kD	TRAPP220	Mediator of RNAPII transcription subunit 1		
HRP2	74 kD	HDGF2, HDGFL2, HDGFRP2	RNAPII transcription regulator, relieves nucleosome-induced barrier to transcription	2.44	2.42

* Fold induction relative to individual protein expression in DTX-sensitive cells (normalized to 1) was calculated by quantitative immunoblotting as described in Materials and Methods.

## Data Availability

Data generated in this study is available from the corresponding author Carlos A. Casiano (ccasiano@llu.edu).

## Supplementary Materials

The following supplementary figures are available online at https://www.mdpi.com/article/10.3390/cells10102723/s1, Figure S1: Characterization of DTX-resistant PCa cell lines, Table S1: List of canonical pathways revealed by ingenuity pathway analysis of the LEDGF/p75 interactome in PC3 cells, Figure S2: Representative ingenuity pathway analysis map showing protein interactions involving members of the LEDGF/p75 IBD-interactome in PC3 cells, Figure S3: Validation of solubilization efficiency in co-immunoprecipitation studies, Figure S4: Transient knockdown of LEDGF/p75, JPO2, menin, or HRP2 decreases the survival of DU145-DR cells, Figure S5: Validation of the knockdown of LEDGF/p75 (A), menin (B), and JPO2 (C) transcript expression in PC3-DR and DU145-DR cells (SD vs. KD) using qPCR, Figure S6: Knockdown of LEDGF/p75, JPO2, menin, or HRP2 inhibits the clonogenicity of DU145-DR cells, Figure S7: LEDGF/p75 silencing has no effect on lineage-specific nuclear steroid receptors, Figure S8: LEDGF/p75 silencing has no effect on the stemness of DTX-resistant cells.
