# Supplementary material for: The LEDGF/p75 Integrase Binding Domain Interactome Contributes to the Survival, Clonogenicity, and Tumorsphere Formation of Docetaxel-Resistant Prostate Cancer Cells
Source: Cells. 2021 Oct 12;10(10):2723. doi: 10.3390/cells10102723 (PMC8534522; doi:10.3390/cells10102723)

Supplementary Fig. S1

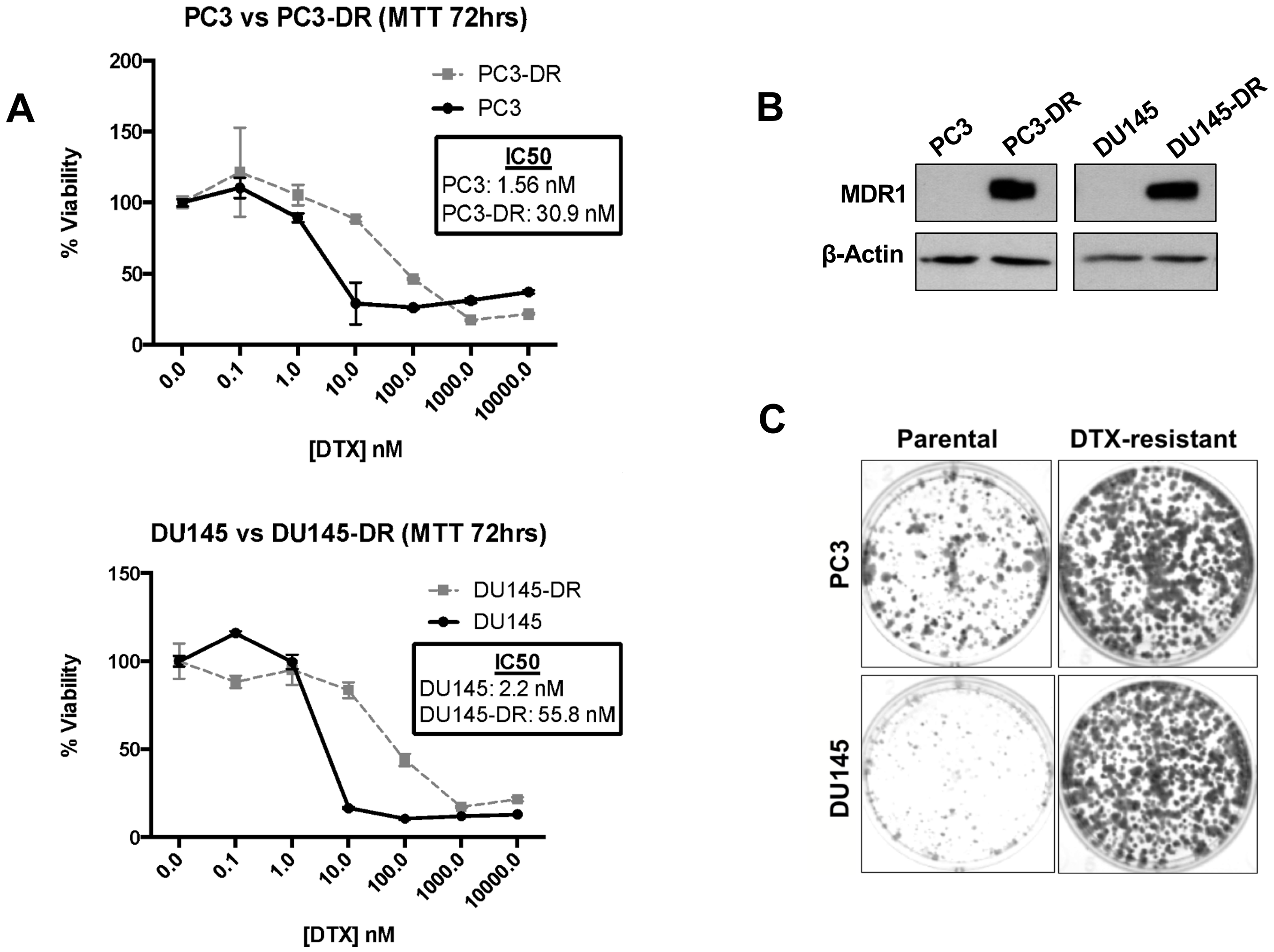

Supplementary Table S1. List of canonical pathways revealed by Ingenuity Pathway Analysis of the LEDGF/p75 interactome in PC3 cells

Canonical Pathways	Associated signaling molecules	Upstream regulators	p-values
p38 MAPK Signaling	ASK1/MAP3K5, MYC	-	9.32e-04
HOTAIR Regulatory Pathway	MLL/KMT2A, MYC	PRC2, HOXD10, MIR7, SETDB1, STAT3	1.7e-03
Endoplasmic Reticulum Stress Pathway	ASK1/MAP3K5	-	8.25e-03
Polyamine Regulation in Colon Cancer	MYC	TCF4-CTNNβ	9.04e-03
Molecular Mechanisms of Cancer	ASK1/MAP3K5, MYC	Smad2/3-Smad4	1.02e-02
Estrogen-mediated S-phase Entry	MYC	-	1.02e-02
4-1BB Signaling in T Lymphocytes	ASK1/MAP3K5	-	1.26e-02
Cardiac Hypertrophy Signaling (Enhanced)	ASK1/MAP3K5, MYC	-	1.54e-02
BAG2 Signaling Pathway	MYC	-	1.68e-02
Myc Mediated Apoptosis Signaling	MYC	-	1.96e-02
CD27 Signaling in Lymphocytes	ASK1/MAP3K5	-	2.07e-02
Unfolded protein response	ASK1/MAP3K5	-	2.19e-02
Endometrial Cancer Signaling	MYC	-	2.34e-02
Induction of Apoptosis by HIV1	ASK1/MAP3K5	-	2.38e-02
Thrombopoietin Signaling	MYC	-	2.46e-02
ErbB2-ErbB3 Signaling	MYC	-	2.54e-02
Cell Cycle: G1/S Checkpoint Regulation	MYC	-	2.61e-02
Small Cell Lung Cancer Signaling	MYC	-	2.77e-02
ERK5 Signaling	MYC	-	2.8e-02
IL-15 Signaling	MYC	STAT5	2.92e-02
Role of MAPK Signaling in Inhibiting the Pathogenesis of Influenza	ASK1/MAP3K5	-	2.92e-02
Neurotrophin/TRK Signaling	ASK1/MAP3K5	-	2.96e-02
IL-7 Signaling Pathway	MYC	-	3.04e-02
Thyroid Cancer Signaling	MYC	TCF/LEF	3.07e-02
Role of MAPK Signaling in the Pathogenesis of Influenza	ASK1/MAP3K5	-	3.11e-02
Prolactin Signaling	MYC	-	3.15e-02
LPS-stimulated MAPK Signaling	ASK1/MAP3K5	-	3.23e-02
FGF Signaling	ASK1/MAP3K5	-	3.27e-02
PDGF Signaling	MYC	-	3.34e-02
Acute Myeloid Leukemia Signaling	MYC	TCF/LEF	3.46e-02
RAK Signaling in Osteoclasts	ASK1/MAP3K5	-	3.46e-02
Death Receptor Signaling	ASK1/MAP3K5	MKK 4/7, JNK1, BCL-2, DIABLO, HtrA2, CYCS, APAF1, Apaf1-Cycs, CASP 9, CASP 7, XIAP, CASP 3, CASP 6, DNA Repair, DFFA, DFFB, DNA fragmentation, Apoptosis, ACIN1, Chromatin condensation, ARHGDIB, ACTIN, Cell shrinkage	3.57e-02
Bladder Cancer Signaling	MYC	MSK1	3.76e-02
Apoptosis Signaling	ASK1/MAP3K5	-	3.88e-02
SAPK/JNK Signaling	ASK1/MAP3K5	-	3.95e-02
Chronic Myeloid Leukemia Signaling	MYC	-	3.99e-02
Mouse Embryonic Stem Pluripotency	MYC	CTNN β, STAT3 dimer	3.99e-02
Sumoylation Pathway	ASK1/MAP3K5	-	3.99e-02
Neuregulin Signaling	MYC	-	4.07e-02
Telomerase Signaling	MYC	-	4.14e-02
Role of MAPK Signaling in Promoting the Pathogenesis of Influenza	ASK1/MAP3K5	-	4.26e-02
Type 1 Diabetes Mellitus Signaling	ASK1/MAP3K5	-	4.29e-02
NGF Signaling	ASK1/MAP3K5	-	4.41e-02
HGF Signaling	ASK1/MAP3K5	-	4.64e-02
14-3-3-mediated Signaling	ASK1/MAP3K5	-	4.9e-02
P2Y Purigenic Receptor Signaling Pathway	MYC	-	4.9e-02

Supplementary Fig. S2

Molecular Mechanisms of Cancer pathway

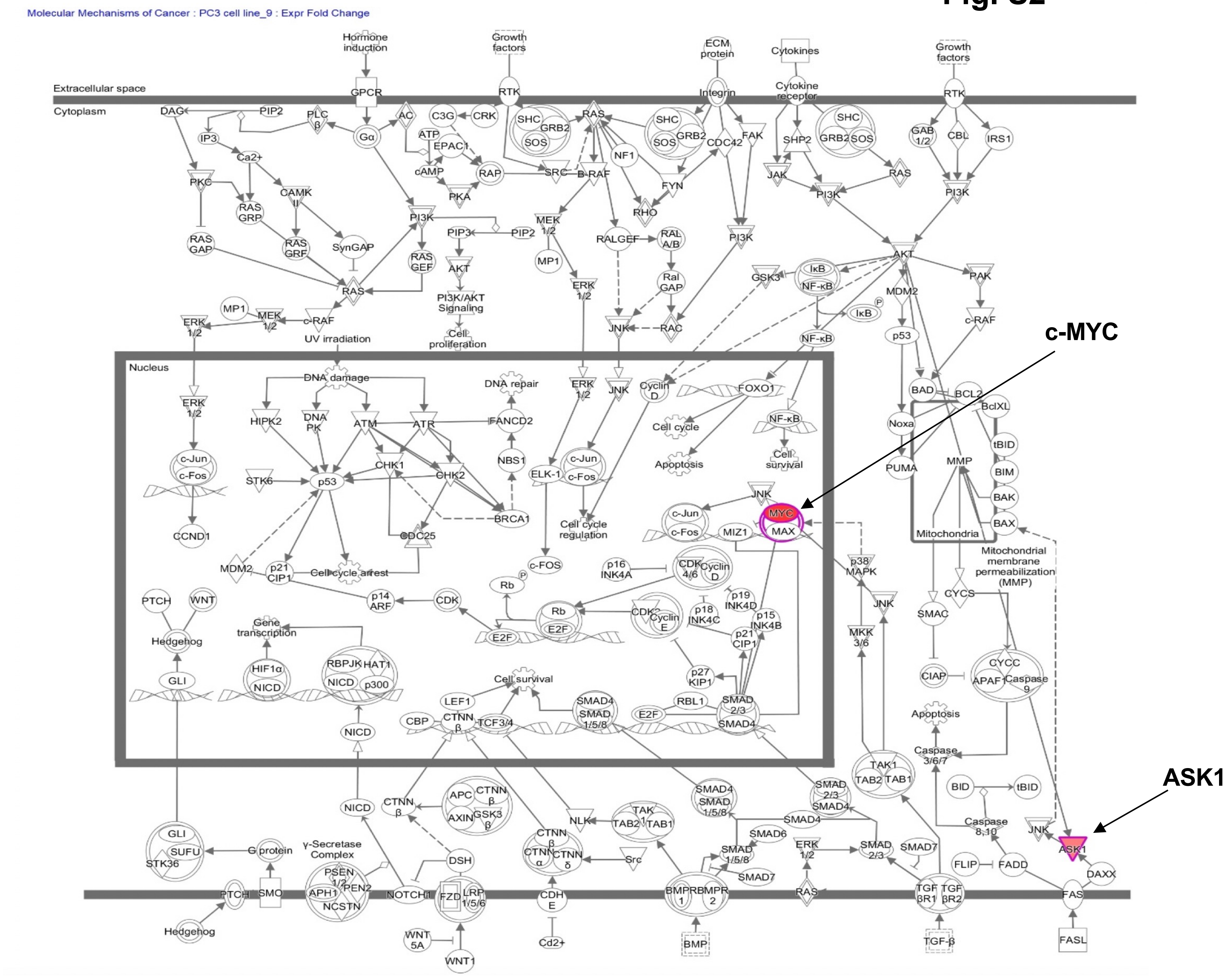

Supplementary Fig. S3

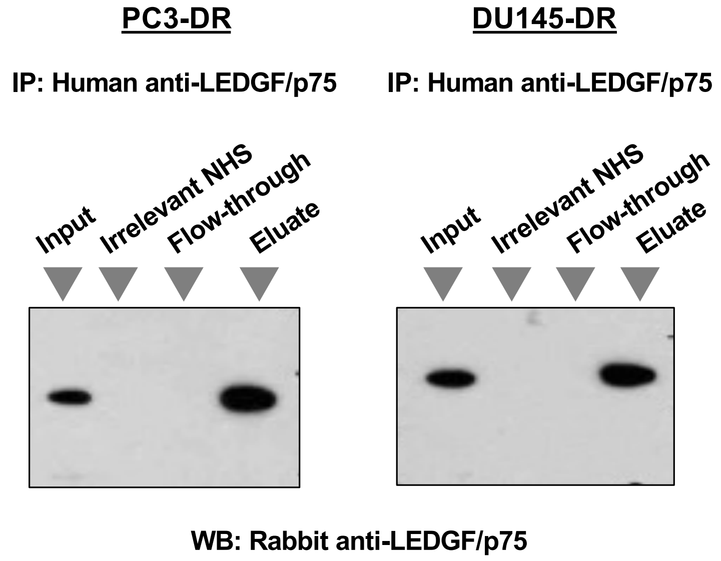

Supplementary Fig. S4

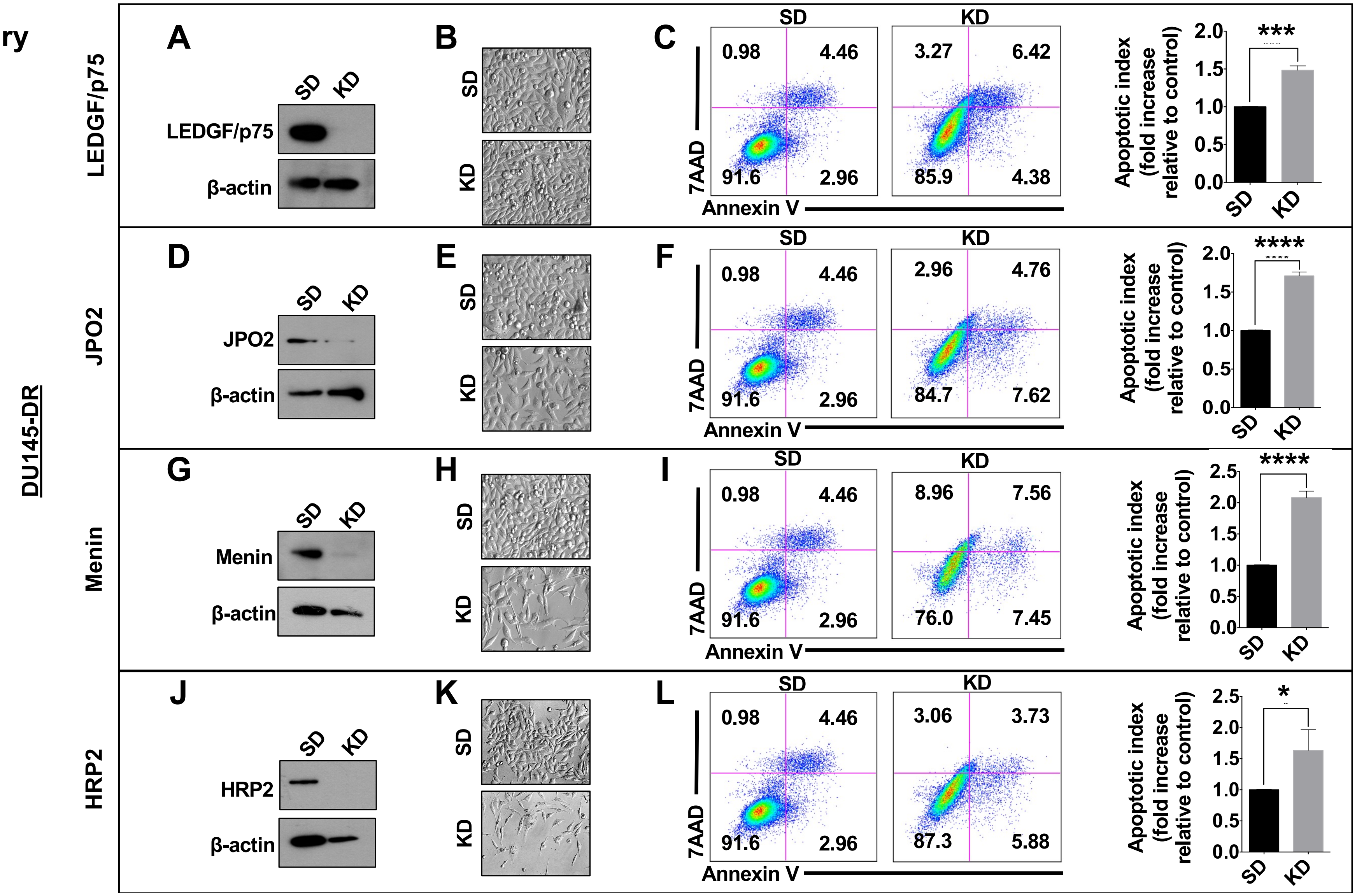

Supplementary Fig. S5

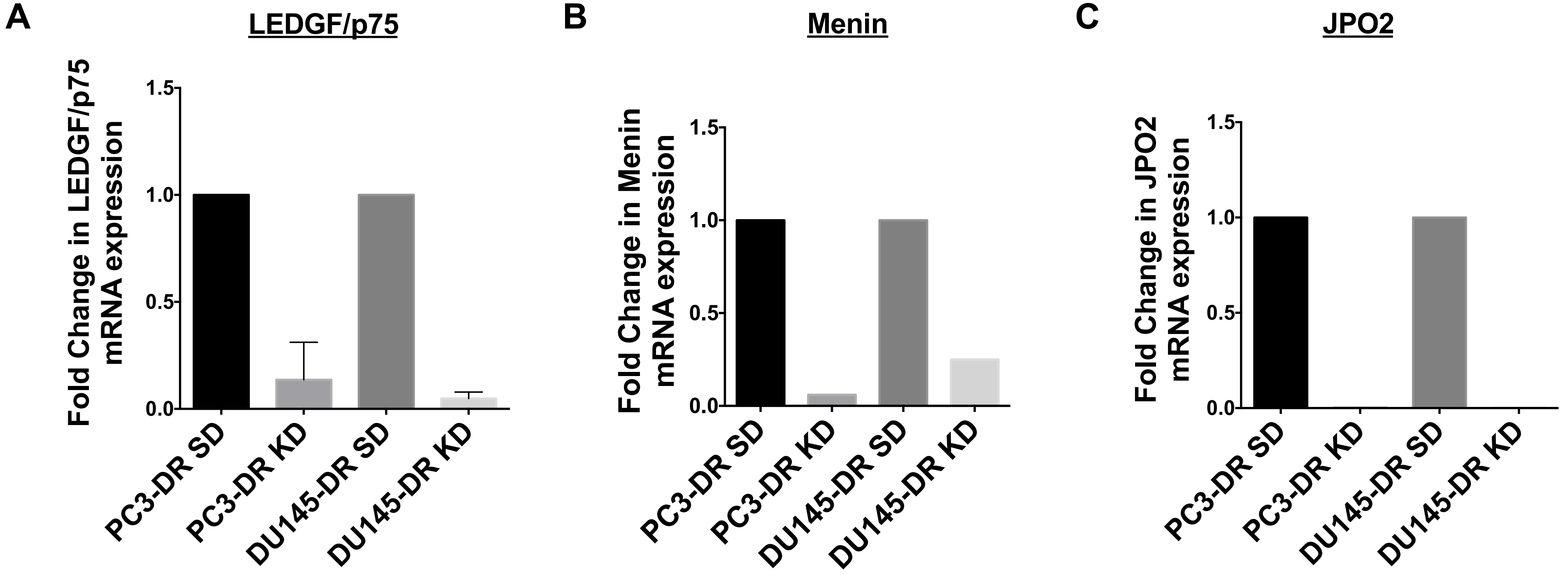

Supplementary Fig. S6

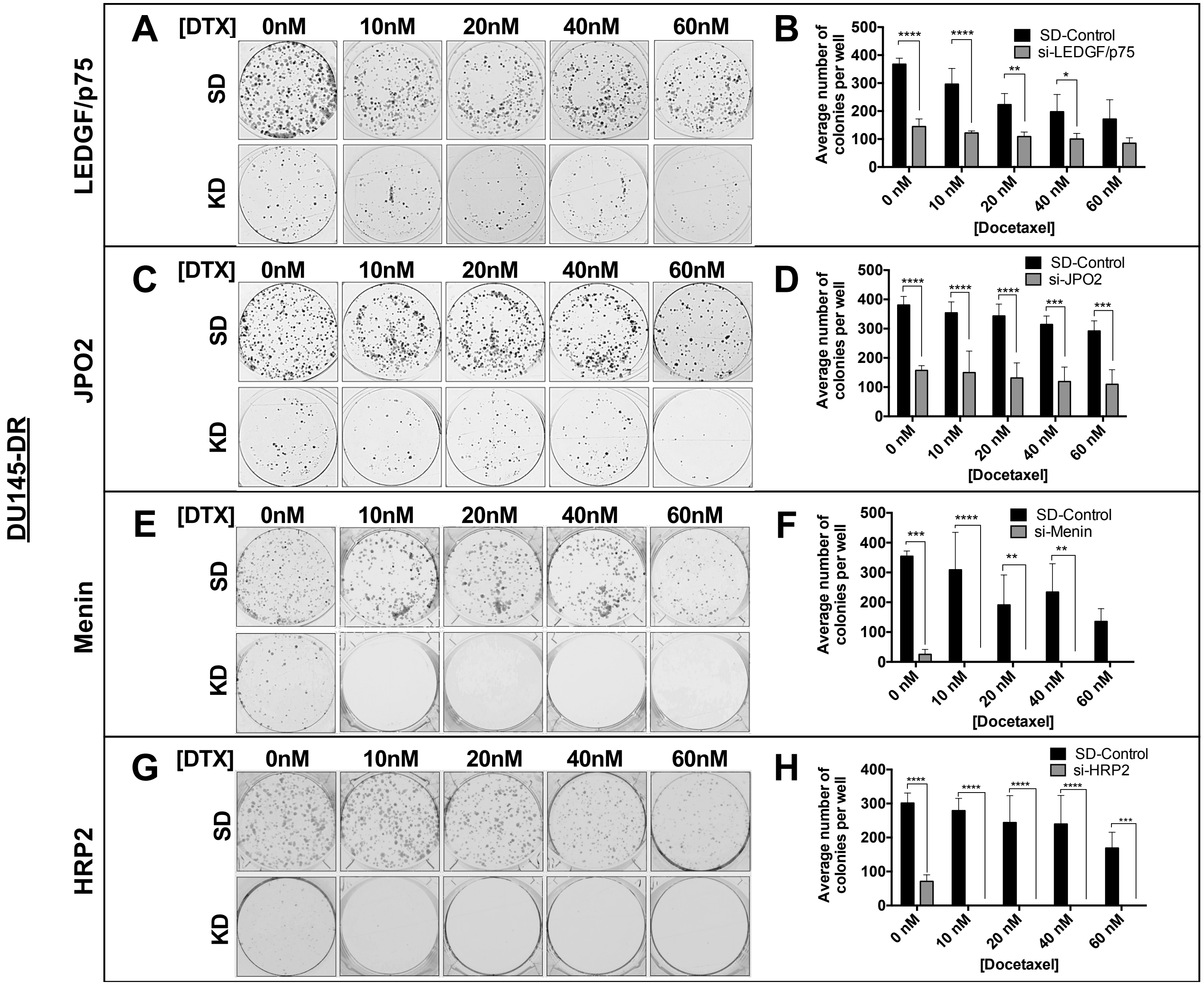

Supplementary
Fig. S7

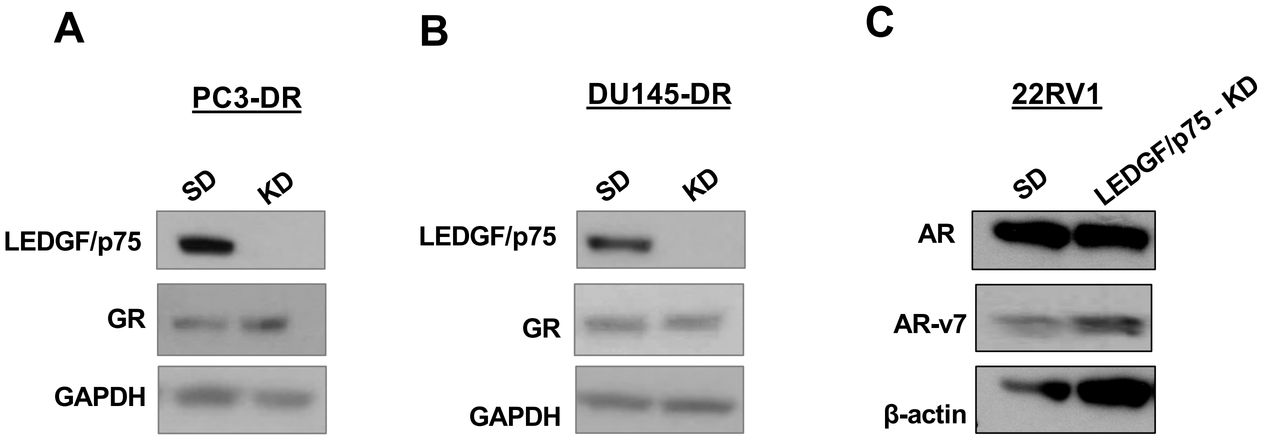

Supplementary Fig. S8

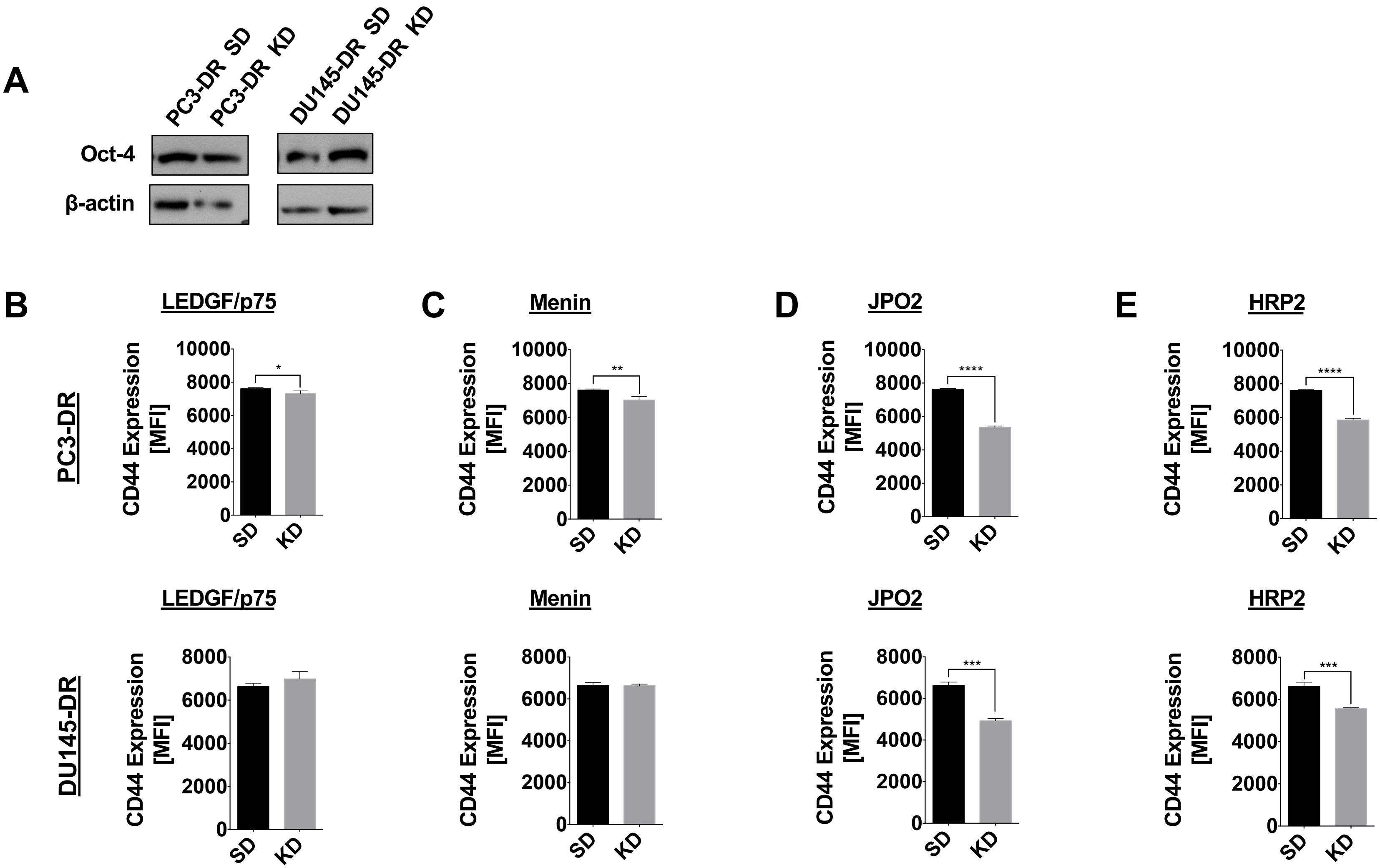

Supplement: Supplementary file 1 [file cells-10-02723-s001.zip › cells-1348272-supplementary.pdf]
